# Loss of *Baiap2l2* destabilizes the transducing stereocilia of cochlear hair cells and leads to deafness

**DOI:** 10.1113/JP280670

**Published:** 2020-11-26

**Authors:** Adam J. Carlton, Julia Halford, Anna Underhill, Jing‐Yi Jeng, Matthew R. Avenarius, Merle L. Gilbert, Federico Ceriani, Kimimuepigha Ebisine, Steve D. M. Brown, Michael R. Bowl, Peter G. Barr‐Gillespie, Walter Marcotti

**Affiliations:** ^1^ Department of Biomedical Science University of Sheffield Sheffield UK; ^2^ Neuroscience Institute University of Sheffield Sheffield UK; ^3^ Oregon Hearing Research Center & Vollum Institute Oregon Health & Science University Portland OR USA; ^4^ Mammalian Genetics Unit MRC Harwell Institute Harwell Campus Oxfordshire UK; ^5^ Oregon Hearing Research Center Oregon Health & Science University Portland OR USA; ^6^ Present address: Department of Pathology Wexner Medical Center The Ohio State University Columbus OH USA; ^7^ Present address: US Army Medical Department Activity‐Korea Camp Humphreys Republic of Korea; ^8^ Present address: UCL Ear Institute University College London London UK

**Keywords:** actin, cochlear, development, hearing loss, mechanoelectrical transduction, mouse, stereocilia

## Abstract

**Key points:**

- Mechanoelectrical transduction at auditory hair cells requires highly specialized stereociliary bundles that project from their apical surface, forming a characteristic graded ‘staircase’ structure.
- The morphogenesis and maintenance of these stereociliary bundles is a tightly regulated process requiring the involvement of several actin‐binding proteins, many of which are still unidentified.
- We identify a new stereociliary protein, the I‐BAR protein BAIAP2L2, which localizes to the tips of the shorter transducing stereocilia in both inner and outer hair cells (IHCs and OHCs).
- We find that *Baiap2l2* deficient mice lose their second and third rows of stereocilia, their mechanoelectrical transducer current, and develop progressive hearing loss, becoming deaf by 8 months of age.
- We demonstrate that BAIAP2L2 localization to stereocilia tips is dependent on the motor protein MYO15A and its cargo EPS8.
- We propose that BAIAP2L2 is a new key protein required for the maintenance of the transducing stereocilia in mature cochlear hair cells.

**Abstract:**

The transduction of sound waves into electrical signals depends upon mechanosensitive stereociliary bundles that project from the apical surface of hair cells within the cochlea. The height and width of these actin‐based stereocilia is tightly regulated throughout life to establish and maintain their characteristic staircase‐like structure, which is essential for normal mechanoelectrical transduction. Here, we show that BAIAP2L2, a member of the I‐BAR protein family, is a newly identified hair bundle protein that is localized to the tips of the shorter rows of transducing stereocilia in mouse cochlear hair cells. BAIAP2L2 was detected by immunohistochemistry from postnatal day 2.5 (P2.5) throughout adulthood. In *Baiap2l2* deficient mice, outer hair cells (OHCs), but not inner hair cells (IHCs), began to lose their third row of stereocilia and showed a reduction in the size of the mechanoelectrical transducer current from just after P9. Over the following post‐hearing weeks, the ordered staircase structure of the bundle progressively deteriorates, such that, by 8 months of age, both OHCs and IHCs of *Baiap2l2* deficient mice have lost most of the second and third rows of stereocilia and become deaf. We also found that BAIAP2L2 interacts with other key stereociliary proteins involved in normal hair bundle morphogenesis, such as CDC42, RAC1, EPS8 and ESPNL. Furthermore, we show that BAIAP2L2 localization to the stereocilia tips depends on the motor protein MYO15A and its cargo EPS8. We propose that BAIAP2L2 is key to maintenance of the normal actin structure of the transducing stereocilia in mature mouse cochlear hair cells.

## Key points

- Mechanoelectrical transduction at auditory hair cells requires highly specialized stereociliary bundles that project from their apical surface, forming a characteristic graded ‘staircase’ structure.
- The morphogenesis and maintenance of these stereociliary bundles is a tightly regulated process requiring the involvement of several actin‐binding proteins, many of which are still unidentified.
- We identify a new stereociliary protein, the I‐BAR protein BAIAP2L2, which localizes to the tips of the shorter transducing stereocilia in both inner and outer hair cells (IHCs and OHCs).
- We find that *Baiap2l2* deficient mice lose their second and third rows of stereocilia, their mechanoelectrical transducer current, and develop progressive hearing loss, becoming deaf by 8 months of age.
- We demonstrate that BAIAP2L2 localization to stereocilia tips is dependent on the motor protein MYO15A and its cargo EPS8.
- We propose that BAIAP2L2 is a new key protein required for the maintenance of the transducing stereocilia in mature cochlear hair cells.

## Introduction

The perception of sound depends on the transduction of acoustic information into electrical signals by the sensory hair cells, which requires the opening of mechanically gated ion channels (Fettiplace & Kim, 2014). The hair bundles of cochlear inner and outer hair cells (IHCs and OHCs) consist of three rows of stereocilia, which are cross‐linked by several types of extracellular links (Tilney *et al*. 1992; Goodyear *et al*. 2005; Vélez‐Ortega & Frolenkov, 2019). In the mammalian cochlea, these mechanoelectrical transducer (MET) channels are located at the tips of the middle and shorter rows of the stereociliary hair bundle that protrude from the apical surface of the hair cells. Stereocilia have a tightly packed and uniformly polarized actin cytoskeletal core (Tilney *et al*. 1992; Bartles, 2000). The length of stereocilia is scaled precisely to form the characteristic staircase‐like structure of the hair bundle (Barr‐Gillespie, 2015). The growth of stereocilia is so tightly regulated that their height within each row is similar not only within a single hair bundle, but also between adjacent bundles, and changes depending on location along the tonotopic axis of the cochlea (Tilney *et al*. 1992; Manor & Kachar, 2008; Petit & Richardson 2009). This indicates a sophisticated level of control over the lengthening and widening of stereocilia, which, in altricial rodents, mainly occurs during late embryonic and early postnatal stages (Roth & Bruns, 1992; Kaltenbach, *et al*. 1994; Zine & Romand, 1996) and involves several actin‐binding proteins and unconventional myosin motors (Barr‐Gillespie, 2015; Vélez‐Ortega & Frolenkov, 2019).

One well characterized mechanism regulating the lengthening of stereocilia involves an extended protein complex, where the motor MYO15A (short isoform: Fang *et al*. 2015) is required to transport the scaffolding protein whirlin (WHRN) (Belyantseva *et al*. 2005) and G‐protein signal modulator 2 (GPSM2) and inhibitory G protein alpha (GNAI) (Tadenev *et al*. 2019) to the stereocilia tips. MYO15A‐whirlin regulation of stereocilia length also requires interaction of the complex with epidermal growth factor receptor substrate 8 (EPS8) (Manor *et al*. 2011). EPS8 is an actin‐regulatory protein with actin‐bundling and cross‐linking activity (Croce *et al*. 2004; Hertzog *et al*. 2010); in the cochlea, it is primarily located at the tips of tallest row of stereocilia in both OHCs and IHCs (Furness *et al*. 2013). In the absence of the protein, the stereocilia of cochlear hair cells fail to elongate, leading to deafness in both mice (Zampini *et al*. 2011) and humans (Behlouli *et al*. 2014). The EPS8 family‐related protein EPS8L2, which is also endowed with actin‐binding activity (Offenhauser *et al*. 2004), is mainly expressed at the tip of the shorter rows of stereocilia. *Eps8l2* knockout mice develop a late‐onset, progressive hearing loss that is caused by the gradual loss of the shorter rows of stereocilia (Furness *et al*. 2013). The exact mechanism by which these proteins are segregated between the rows of stereocilia, and whether additional key proteins are involved in this segregation, is currently unknown.

The I‐BAR (inverse bin‐amphiphysin‐Rvs) protein family consists of five members: IRSp53 (also known as BAIAP2), IRTKS (BAIAP2L1), FLJ22582 (BAIAP2L2 or Pinkbar), MIM (MTSS1) and ABBA (MTSS1L) (Ahmed *et al*. 2010). I‐BAR proteins are generally able to detect and induce negative membrane curvature (Zhao *et al*. 2011) by dimerizing and binding to acidic phospholipids (Ahmed *et al*. 2010) via their N‐terminal BAR domain. All members of the family also possess a C‐terminal WH2 domain (Wiskott‐Aldrich homology 2: Zhao *et al*. 2011), which binds globular actin (Lee *et al*. 2007, Saarikangas *et al*. 2008). In addition, BAIAP2, BAIAP2L1 and BAIAP2L2 possess a central SH3 domain (Ahmed *et al*. 2010), which is capable of recruiting actin effector proteins including EPS8 (Postema *et al*. 2018), mDia1 (Goh *et al*. 2012) and WAVE2 (Suetsugu *et al*. 2006). The ability of I‐BAR proteins to localize EPS8 in the gut to regulate actin based protrusions, such as microvilli growth (Postema *et al*. 2018) and filopodia dynamics (Sudhaharan *et al*. 2016), suggests that the I‐BAR proteins may possess a role in stereocilia morphogenesis and/or maintenance via EPS8 targeting. In particular, BAIAP2L2 binds to actin via its C‐terminal WH2 domain (Lee *et al*. 2007) and it has been identified as a hair cell protein using RNA‐seq (Scheffer *et al*. 2015; Zhu *et al*. 2019), as well as a novel hearing loss gene in mice (Bowl *et al*. 2017). A recent genome‐wide association study has also provided a link between *Baiap2l2* and hearing pathology in humans (Wells *et al*. 2019), indicating the crucial role of this gene in hearing function.

In the present study, we generated two *Baiap2l2* knockout mouse lines and investigated the structure and physiology of the stereociliary bundles of cochlear hair cells. In the absence of BAIAP2L2, the hair bundles of OHCs and IHCs progressively lose their transducing stereocilia rows, leading to a rapid increase of hearing thresholds. By 8 months of age, *Baiap2l2* deficient mice are deaf. The basolateral membrane characteristics of the hair cells were not affected by absence of BAIAP2L2. We also found that BAIAP2L2 is specifically localized at the tip of the shorter rows of stereocilia, especially row 2, in cochlear hair cells from just after birth up to adult ages in mice. We showed that BAIAP2L2 interacts with CDC42, RAC1, EPS8 and ESPNL, comprising proteins that are essential for the normal morphogenesis and maintenance of the stereociliary bundles. Moreover, the localization of BAIAP2L2 to the stereocilia tip requires both the acting‐regulator protein EPS8 and the motor protein MYO15A. We propose that MYO15A, EPS8 and BAIAP2L2 are part of a stereociliary protein complex involved in the maintenance of adult cochlear hair cells transducing stereocilia.

## Methods

### Ethical statement

In the UK, all animal work was performed at the University of Sheffield (UK), as licensed by the Home Office under the Animals (Scientific Procedures) Act 1986 (PPL_PCC8E5E93) and approved by the University of Sheffield Ethical Review Committee (180 626_Mar). For *in vitro* experiments, mice were killed by cervical dislocation followed by decapitation. For *in vivo* auditory brainstem responses (ABRs) and distortion product otoacoustic emissions (DPOAEs), mice were anaesthetized using an i.p. injection of ketamine (100 mg kg^−1^ body weight; Fort Dodge Animal Health, Fort Dodge, IA, USA) and xylazine (10 mg kg^−1^, Rompun 2%; Bayer HealthCare LLC, Tarrytown, NY, USA). At the end of the *in vivo* recordings, mice were either culled by cervical dislocation or recovered from anaesthesia with an i.p. injection of atipamezole (1 mg kg^−1^). Mice under recovery from anaesthesia were returned to their cage, placed on a thermal mat and monitored over the following 2–5 h.

In the USA, all animal procedures were approved by the Institutional Animal Care and Use Committee (IACUC) at Oregon Health & Science University (protocol IP00000714). For *in vitro* experiments, neonatal mice were killed by cervical dislocation followed by decapitation.

In all cases, experiments were carried out according to the guidelines laid down by the University of Sheffield and Oregon Health & Science University animal welfare committees, and conform to the principles and regulations as described in the editorial by Grundy (2015).

### Characterization of *Baiap2l2^tm1b^* mice

The *Baiap2l2^tm1a^* allele (EM:0 7678) was imported from the EMMA repository (Orleans, France) to the MRC Harwell Institute (Oxfordshire, UK), as licensed by the Home Office under the Animals (Scientific Procedures) Act 1986 (PPL_PBF9BD884) and approved by the local Ethical Review Board. To obtain *Baiap2l2^tm1b^* animals, cre‐mediated conversion of the ‘knockout‐first’ tm1a allele was achieved by treating IVF derived embryos with a cell permeable cre‐enzyme (Excellgen, Derwood, MD, USA). *Baiap2l2^tm1b^* mice were generated and maintained on the C57BL/6N background strain, and genotyped using the primers: wild‐type forward primer 5′‐CAG ATC CTC AAC ACC AAC GA‐3′; knockout forward primer 5′‐CCA GTT GGT CTG GTG TCA‐3′; reverse primer 5′‐TCG GCT CCT TGA TAA AAT GG‐3′.

### Production of the *Baiap2l2^Δ16^* ^^mutant using CRISPR (clustered regularly interspaced short palindromic repeats)

The *Baiap2l2* locus was targeted for CRISPR‐mediated knockout using guide RNAs (gRNAs) designed to exons 4 and 10 (http://crispr.mit.edu/). The gRNA sequences (exon 4, 5′‐GCGGCACTTGAACTCAGAC; exon 10, 5′‐CAATTCCTTCGGCGAGCGCC) were individually cloned into the DR274 gRNA expression vector (#42 250; Addgene, Watertown, MA, USA) and transcribed using the MegaScript T7 kit (ThermoFisher, Waltham, MA, USA). The *in vitro* transcribed gRNAs were purified using the NucleoSpin miRNA kit (Macherey‐Nagel, Düren, Germany) and quantified using a NanoDrop spectrophotometer (ThermoFisher). A mixture containing each gRNA (30 ng μl^−1^) and the Cas9 mRNA (110 ng μl^−1^) (Trilink, San Diego, CA, USA) was prepared, injected into zygotes and implanted into pseudopregnant females. Founders were screened for mutations in the targeted exons, as well as for large deletions in the intervening sequence. An out‐of‐frame mutation in the coding region of exon 4 (c.247_262delCGGCACTTGAACTCAG) that resulted in a premature termination codon (p.Arg83ThrfsTer67) was identified, backcrossed on to the C57BL/6 background and propagated for the experiments described in the present study.

*Baiap2l2* CRISPR mice (denoted *Baiap2l2^Δ16^*) were genotyped using the following primers: wild‐type forward primer 5′‐CCA GCG GCA CTT GAA CTC AG‐3′; wild‐type reverse primer 5′‐CTG AGA CTC GGC TCC TTG AT‐3′; knockout forward primer 5′‐GGT TCA GAG CAT CAT GGA GC‐3′; knockout reverse primer 5′‐ACC TCC AGG TCT GGG TGT GT‐3′.

### Tissue preparation

Patch clamp recordings were performed from hair cells within the 9–12 kHz region of the cochlear apical coil (Müller *et al*. 2005). The apical coil of wild‐type (C57BL/6) and *Baiap2l2^tm1b^* mice was dissected out in extracellular solution composed of (in mm): 135 NaCl, 5.8 KCl, 1.3 CaCl_2_, 0.9 MgCl_2_, 0.7 NaH_2_PO_4_, 5.6 d‐glucose and 10 Hepes‐NaOH. Sodium pyruvate (2 mm), amino acids and vitamins were added from concentrates (Thermo Fisher Scientific, Cambridge, UK). The pH adjusted to 7.48 with 1 m NaOH (∼308 mmol kg^−1^). The dissected apical coil was then transferred to a microscope chamber and immobilized via a nylon mesh attached to a stainless steel ring. The chamber (volume 2 mL) was perfused from a peristaltic pump and mounted on the stage of an upright microscope (BX51, Olympus, Tokyo, Japan; DMLFS, Leica, Wetzlar, Germany) with Nomarski differential interference contrast (DIC) optics (63× or 60× water immersion objective) and a 15× eyepiece. The microscope chamber was continuously perfused with extracellular solution via a peristaltic pump (Cole‐Palmer, Eaton‐Socon, UK).

### Whole‐cell electrophysiology

IHCs and OHCs were studied in acutely dissected organs of Corti from postnatal day 6 (P6) to P40. Patch clamp recordings were performed at room temperature (20–24°C) using an Optopatch amplifier (Cairn Research Ltd, Faversham, UK) as described previously (Furness *et al*. 2013; Ceriani *et al*. 2019; 2020; Jeng *et al*. 2020*ab*). Patch pipettes were pulled from soda glass capillaries and had a typical resistance in extracellular solution of 2–3 MΩ. To reduce the electrode capacitance, patch electrodes were coated with surf wax (Mr Zoggs SexWax; SexWax, Inc., Carpinteria, CA, USA). The patch pipette intracellular solution contained (in mm): 131 KCl, 3 MgCl_2_, 1 EGTA‐KOH, 5 Na_2_ATP, 5 Hepes‐KOH and 10 Na‐phosphocreatine (pH was adjusted with 1 m KOH to 7.28; 294 mmol kg^−1^). Data acquisition was controlled by pClamp software using Digidata 1440A (Molecular Devices, San Jose, CA, USA). Recordings were low‐pass filtered at 2.5 kHz (8‐pole Bessel), sampled at 5 khz and stored on a computer for off‐line analysis (Clampfit, Molecular Devices; Origin 2020: OriginLab, Northampton, MA, USA). For voltage‐clamp experiments, membrane potentials were corrected off‐line for the residual series resistance *R*_s_ after compensation (usually 80%: IHCs 1.2 ± 0.2 MΩ, *n* = 25; OHCs 1.0 ± 0.1 MΩ, *n* = 21) and the liquid junction potential (LJP) of −4 mV, which was measured between electrode and bath solutions. Holding currents were plotted as zero current to allow a better comparison between recordings. Voltage clamp protocols are referred to a holding potential of −84 mV or −64 mV depending on the protocol used. Voltage recordings in current clamp were also corrected for the LJP.

For mechanoelectrical transducer (MET) current recordings, the hair bundles of hair cells were displaced using a fluid jet from a pipette driven by a 25 mm diameter piezoelectric disc (Corns *et al*. 2014; Marcotti *et al*. 2016; Corns *et al*. 2018). The pipette was pulled from borosilicate glass to a final overall length of 5.3–5.5 cm. The fluid jet pipette tip had a diameter of 8–10 μm and was positioned near the hair bundles to elicit a maximal MET current. Mechanical stimuli were applied as 50 Hz sinusoids (filtered at 1 kHz, 8‐pole Bessel) with driving voltages of up to ± 40 V for OHCs and ±20 V for IHCs. Prior to the positioning of the fluid jet by the hair bundles, any steady‐state pressure was removed by monitoring the movement of debris in front of the pipette. The use of the fluid‐jet allows for the efficient displacement of the hair bundles in both the excitatory and inhibitory directions, which is essential for performing reliable measurements of the resting open probability of the MET channels (Corns *et al*. 2014; Marcotti *et al*. 2016; Corns *et al*. 2018).

### Auditory brainstem responses

Following the onset of anaesthesia (see Ethics statement above) and the loss of the retraction reflex with a toe pinch, mice were placed in a soundproof chamber (MAC‐3 acoustic chamber; IAC Acoustic, Chandlers Ford, UK). Both male and female mice were placed on a heated mat (37°C) with the animal's pinna being positioned at a distance of 10 cm from the loudspeaker. Two subdermal electrodes were placed under the skin behind the pinna of each ear (reference and ground electrode), with one electrode half‐way between the two pinna on the vertex of the cranium (active electrode) as described previously (Ingham *et al*. 2011). Sound stimuli were delivered to the mouse ear by a loudspeaker (MF1‐S, Multi Field Speaker; Tucker‐Davis Technologies, Alachua, FL, USA), which was calibrated with a low‐noise microphone probe system (ER10B+; Etymotic Research Inc., Elk Grove Village, IL, USA). Experiments were performed using a customized software (Ingham *et al*. 2011) driving an RZ6 auditory processor (Tucker‐Davis Technologies). ABR thresholds, which were delivered as broad‐band clicks and pure tone stimuli of frequencies at 3, 6, 12, 18, 24, 30 and 36 kHz, were defined as the lowest sound level where any recognizable feature of the waveform was visible. Stimulus sound pressure levels were up to 95 dB SPL, presented in steps of 5 dB SPL (average of 256 repetitions). Tone bursts were 5 ms in duration with a 1 ms on/off ramp time presented at a rate of 42.6 s^–1^.

Wave 1 amplitude and latency were measured from ABR recordings obtained by stimulating mice with a pure tone (12 kHz). We selected the 12 kHz value because it is close to the frequency range used for the *in vitro* work. An initial automatic identification of Wave 1 was carried out using a custom software routine based on the *find_peaks* function of the scipy.signal Python module (Python 3.7; Python Software Foundation, Wilmington, DE, USA) (Virtanen *et al*. 2020). The results were manually reviewed and, if required, adjusted to the correct peak. The Wave 1 amplitude was calculated as the difference between the amplitude of the first peak and the first trough of the ABR waveform; the latency was calculated as the delay of the Wave 1 peak from the beginning of the recording. Because the distance of the speaker from the animal is 10 cm (see above), this leads to a delay in the signal of ∼0.3 ms.

### Distortion product otoacoustic emissions

DPOAEs were used to assess OHC function *in vivo* by the synchronous presentation of two stimulus tones (primaries f1 and f2). DPOAEs were recorded at 2f1‐f2 in response to primary tones f1 and f2, where f2/f1 = 1.2. The f2 level (L2) was set from 20 to 80 dB (maximum level set for our system) in 10 dB increments, and the f1 level (L1) was set equal to L2. Frequency pairs of tones between f2 = 6.5 kHz and f2 = 26.3 kHz were presented directly into the left ear canal of mice by means of a coupler, which was connected to two calibrated loudspeakers using 3 cm plastic tubes (MF1‐S, Multi Field Speaker; Tucker‐Davis Technologies).

Recordings were performed in a soundproof chamber (MAC‐3 Acoustic Chamber; IAC Acoustic) and the emission signals were recorded by a low‐noise microphone (ER10B+: Etymotic Research Inc.) connected to the coupler described above. Experiments were performed using BioSigRZ software driving an RZ6 auditory processor (Tucker‐Davis Technologies). The DPOAE thresholds were defined by the minimal sound level where the DPOAEs were above the SD of the noise. The determined DPOAE thresholds were plotted against the geometric mean frequency of f1 and f2. Stimulus sound pressure levels were up to 80 dB SPL, presented in steps of 10 dB. The response signal was averaged over 500 repetitions.

### Scanning electron microscopy (SEM)

For SEM, the dissected mouse cochleae were initially fixed by a very gentle intralabyrinthine perfusion using a 10 μL pipette tip through the round window. The fixative contained 2.5% v/v glutaraldehyde in 0.1 m sodium cacodylate buffer plus 2 mm Ca_2_Cl (pH 7.4). After a few minutes, the cochleae were immersed in the above fixative for 2 h at room temperature. After the fixation, the organ of Corti was exposed by removing the bone from the apical coil to the cochlea and then immersed in 1% osmium tetroxide in the cacodylate buffer for 1 h. For osmium impregnation, which avoids gold coating, cochleae were incubated in solutions of saturated aqueous thiocarbohydrazide (20 min) alternating with 1% osmium tetroxide in buffer (2 h) twice (the OTOTO technique: Furness & Hackney, 1986). The cochleae were then dehydrated through an ethanol series and critical point dried using CO_2_ as the transitional fluid (EM CPD300; Leica) and mounted on specimen stubs using conductive silver paint (Agar Scientific, Stansted, UK). The apical coil of the organ of Corti was examined at 10 kV using a Vega3 LMU scanning electron microscope (Tescan, Brno, Czechia).

### Immunofluorescence microscopy

In the UK, the inner ears were dissected and fixed with 4% paraformaldehyde in PBS (pH 7.4) for 20 min at room temperature. Cochleae were washed three times in PBS for 10 min and fine dissected. Samples were incubated in PBS supplemented with 5% normal goat or horse serum and 0.5% Triton X‐100 for 1 h at room temperature. The samples were immunolabelled with primary antibodies overnight at 37˚C, washed three times with PBS and incubated with the secondary antibodies for 1 h at 37˚C. Antibodies were prepared in 1% serum and 0.5% Triton X‐100 in PBS. F‐actin was stained with Texas Red‐X phalloidin (dilution 1:400, ThermoFisher, T7471) within the secondary antibody solution. Primary antibodies were: rabbit‐IgG anti‐BAIAP2L2 (dilution 1:500, Atlas Antibodies, HPA003043), mouse‐IgG1 anti‐EPS8 (dilution 1:1000; 610 143; BD Biosciences, San Jose, CA, USA), mouse‐IgG1 anti‐BK channel (dilution 1:200; 75–408; NeuroMab, Davis, CA, USA), mouse‐IgG1 anti‐CtBP2 (dilution 1:50; 612 044; BD Biosciences), goat‐IgG anti‐ChAT (dilution 1:500; AB144P; Millipore, Burlington, MA, USA), rabbit‐IgG anti‐KCNQ4 (dilution 1:100; SMC‐309; StressMarq Biosciences, Victoria, BC, Canada), mouse‐IgG1 anti‐MYO7a (dilution 1:100; #138‐1s; Developmental Studies Hybridoma Bank, Iowa City, IA, USA), rabbit‐IgG anti‐MYO7a (dilution 1:500; 25–6790; Proteus Biosciences, Ramona, CA, USA) mouse‐IgG2a anti‐PSD95 (dilution 1:1000; MABN68; Millipore), rabbit‐IgG anti‐prestin (dilution 1:1000; kindly provided by Robert Fettiplace, University of Wisconsin‐Madison, USA) and rabbit‐IgG anti‐SK2 (dilution 1:500; P0483; Sigma‐Aldrich, St Louis, MO, USA). Secondary antibodies were species appropriate Alexa Fluor or Northern Lights secondary antibodies. Samples were mounted in Vectashield (H‐1000; Vector Laboratories, Inc., Burlingame, CA, USA). The images from the apical cochlear region (8‐12 kHz) were captured with an A1 confocal microscope (Nikon, Tokyo, Japan) equipped with Nikon CFI Plan Apo 60× Oil objective or a LSM 880 AiryScan (Zeiss, Oberkochen, Germany) equipped with a Plan‐Apochromat 63× Oil DIC M27 (Zeiss) objective for super‐resolution images of hair bundles. Both microscopes are part of the Wolfson Light Microscope Facility at the University of Sheffield. Image stacks were processed with Fiji ImageJ (https://imagej.net/Fiji).

In the USA, inner ears were isolated from *Baiap2l2* or *Myo15a^sh2 ^*mice and wild‐type or heterozygote littermates at the indicated ages and dissected in cold Leibovitz's L‐15 medium (L‐15) supplemented with 5 mm Hepes (pH 7.4). Small openings were made within the periotic bones to allow perfusion of the fixative and the ears were then fixed in 4% formaldehyde in PBS for 1 h at room temperature. Inner ears were washed in PBS, then the cochleae were dissected out from the periotic bone and the lateral wall was removed. Cochleae were permeabilized in 0.5% Triton X‐100 in PBS for 10 min at room temperature. For stereocilia dimension analysis of *Baiap2l2 *mice using only phalloidin, inner ears were then incubated with 0.4 U mL^−1^ Alexa Fluor 488 phalloidin in PBS for 3 h at room temperature. Organs were washed three times in PBS over 15 min and then mounted with Vectashield. For immunolabelling, after permeabilization, samples were blocked for 1 h in PBS supplemented with 5% normal donkey serum. Samples were then incubated overnight in the primary antibody solution and washed three times in PBS over 15 min. The samples were then incubated in secondary antibody containing solution (donkey anti‐rabbit Alexa Fluor 488, donkey anti‐mouse Alexa Fluor 568) with phalloidin (0.4 U mL^−1^ CF568 phalloidin or 0.8 U mL^−1^ CF405 phalloidin) for 3 h at room temperature, washed three times in PBS over 15 min, and mounted in Vectashield. Primary antibodies included rabbit anti‐BAIAP2L2 (dilution 1:200; ab224323; Abcam, Cambridge, MA, USA); mouse anti‐EPS8 (dilution 1:200; #610 143; BD Biosciences); and rabbit anti‐ESPNL (dilution 1:250; Ebrahim *et al*. 2016). All samples were imaged with a 63×, 1.4 NA Plan‐Apochromat objective on a Zeiss Elyra PS.1/LSM710 system equipped with an Airyscan detector and ZEN 2012 (black edition, 64‐bit software; Zeiss) acquisition software. The full‐width at half‐maximum of the point‐spread function (PSF) in the *x*–*y* plane was 186 nm; resolution in the *z*‐axis is ∼3‐fold poorer (Krey *et al*. 2020).

### Measurements of stereocilia dimensions

Phalloidin‐stained cochleae were imaged such that hair bundles were aligned with the *z*‐axis of the stack, and that *x*–*z* reslices showed pairs of stereocilia in profile. Measurements of stereocilia length and width were approximated from stereocilia profiles using Fiji. Reslices were processed for this function by first applying an unsharp mask with a radius of 3 pixels and a mask weight of 0.6, then binarizing the filtered images with a threshold set such that the stereocilia would be discrete. The Analyze Particles function was applied to the resulting binary mask; Show Ellipses was selected, and Fit ellipse was selected in Set Measurements. The major and minor axes of ellipses fit to the processed stereocilia profiles correspond to approximate stereocilia length and width. Because these measurements are deconvolved with the PSF of an objective, they are approximations of stereocilia dimensions rather than absolute dimensions with errors of ∼40% for width and ∼15% for length (Krey *et al*. 2020). However, the measurements obtained as described nonetheless demonstrate the relative changes in stereociliary shape that occur throughout development.

### FM1‐43 uptake

Inner ears were isolated from *Baiap2l2^Δ16 ^*mice and wild‐type or heterozygote littermates at P8.5, and cochleae were dissected in 155 mm NaCl, 6 mm KCl and 3 mm d‐glucose supplemented with 5 mm Hepes, pH 7.3 (low Ca^2+^ saline). In our laboratory, nominally Ca^2+^‐free saline contains 1–2 μm Ca^2+^ as measured by atomic absorption (Kachar *et al*. 2000). Two to three animals per genotype were used for each experiment. Intact cochleae were left on the modiolus, with the stria vascularis removed; as measured by FM1‐43 loading, transduction persisted for tens of minutes under these conditions. Cochleae were kept on ice in low Ca^2+^ saline prior to pulsing with 6 μm FM1‐43 in low Ca^2+^ saline for 30 s with agitation to allow dye loading through the apical surface. Under these conditions, FM1‐43 labelling measures MET current and not membrane cycling (Meyers *et al*. 2003). To control for non‐specific dye uptake, one cochlea per animal was incubated on ice in low Ca^2+^ saline supplemented with 5 mm BAPTA to break tip‐links and prevent dye entry through the MET channel. After pulsing with FM1‐43, organs were washed three times in low Ca^2+^ saline (1–2 min per wash) and fixed in 4% formaldehyde in low Ca^2+^ saline for 20 min. Fixed organs were washed three times (1–2 min per wash) in PBS, mounted in Vectashield, and imaged immediately with a 63×, 1.4 NA Plan‐Apochromat objective on the same system described above (USA laboratory). z‐stacks for one or two fields of view per animal were acquired to capture the apical surface of the hair cells and 2–5 μm deep. To quantify differences in FM1‐43 uptake across conditions (with or without BAPTA) and genotypes (*Baiap2l2^Δ16^*/+, *Baiap2l2^Δ16/Δ16^*), the signal intensity was measured in Fiji from resions of interest drawn in slices just below (∼0.5 μm) the apical surface. FM1‐43 uptake was variable across experiments, so single cell intensities were normalized to the average wild‐type control (no BAPTA) signal.

### Bacterial expression and characterization of recombinant proteins for pull‐down assays

BAIAP2L2 domains (N‐terminal, amino acids 1–261; C‐terminal, amino acids 262–522; SH3, amino acids 323–386) were expressed in *Escherichia coli* BL21(DE3) with an N‐terminal GST tag using the pGEX‐4T3 vector (GE Healthcare Life Sciences, Chicago, IL, USA). EPS8, EPS8L1, EPS8L2, ESPNL, CDC42, RAC1 were expressed in in *Escherichia coli* BL21(DE3) with N‐terminal 6xHis‐2xHA tags using a modified pTrcHis2‐C vector (ThermoFisher). To map the binding site for these proteins on BAIAP2L2, constructs of truncated BAIAP2L2 C‐terminal domains, with (amino acids 323–522) and without the SH3 domain (amino acids 387–522), were made. Protein expression was induced with the addition of 0.5 mm isopropyl β‐d‐1‐thiogalactopyranoside, and continued overnight at 25°C. Bacterial extracts were prepared under non‐denaturing conditions by first sonicating cells resuspended in PBS supplemented with protease inhibitor cocktail (Sigma‐Aldrich), and then clarifying the sonicated lysates by centrifugation at 12 000 ***g*** for 30 min. Supernatants (clarified bacterial extracts) containing soluble protein were collected, and expression levels were assessed by immunoblotting. For pull‐down assays, GST‐tagged BAIAP2L2 domains were used as bait proteins. Glutathione Sepharose 4B beads (GE Healthcare Life Sciences) were loaded with GST‐tagged proteins by incubation with clarified bacterial extracts for 1 h at room temperature with rotation followed by three washes in PBS (5 min at 500 ***g***). Loaded beads were then incubated with bacterial extracts containing 6xHis‐2xHA tagged prey proteins for 1 h at room temperature with rotation to allow complexes to form. Unbound proteins were washed off (three times for 5 min at 500 ***g***) with PBS. Beads with bound complexes were transferred to spin columns, and incubated for 10 min in hot 2× LDS (ThermoFisher) sample buffer before spinning for 5 min at 1000 ***g*** to elute into 2× reducing agent (Thermo Fisher Scientific). Eluted samples (20% of total eluate) and input lysates (1% of sample applied to beads) were analysed by immunoblotting and detected with either rabbit anti‐GST or mouse anti‐6xHis.

### Statistical analysis

Statistical comparisons of means were made by Student's two‐tailed *t* test or, for multiple comparisons, analysis of variance (one‐way or two‐way ANOVA followed by a suitable post test). *P* < 0.05 was considered statistically significant. Only mean values with a similar variance between groups were compared. Average values are quoted in text and figures as the mean ± SD. For DPOAE experiments, as a result of the presence of ‘not found’ values (i.e. above the upper threshold limit of our equipment), the non‐parametric aligned ranks transformation two‐way ANOVA statistical test (Mann–Whitney *U* test for pairwise comparisons with Bonferroni adjusted *P* values) was used; data are quoted as median, as well as the first and third quartiles (see also: (Jeng *et al*. 2020b).

## Results

### Generation of *Baiap2l2* knockout mice

**Figure 1:**
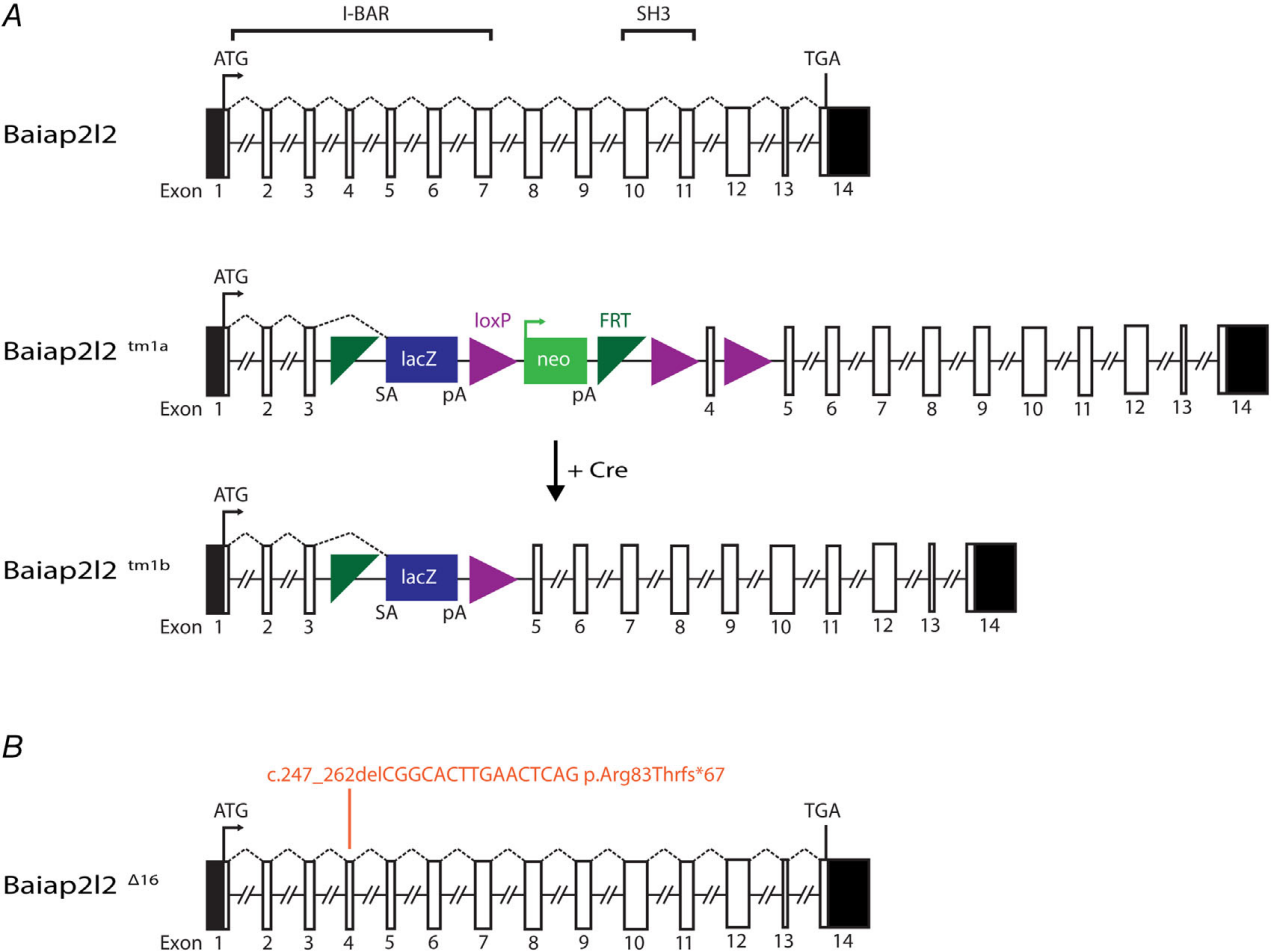
Schematic representation of the genomic structure of the mouse *Baiap2l2* *A*, *BAI1‐associated protein 2‐like 2* (*Baiap2l2*) gene (ENSMUSG00000018126; MGI:2 652 819) comprises 14 exons, spanning ∼30 kb of genomic DNA on chromosome 15. BAIAP2L2 is a 522 amino acid phosphoinositide‐binding protein that contains an I‐BAR domain (encoded by exons 1 to 7) and an SH3 domain (encoded by exons 10 and 11). The ATG (translation start) and the TGA (Stop) sites are in exons 1 and 14, respectively, and the untranslated regions are shown in black. The IMPC uses different targeting strategies to produce knockout alleles, which rely on the identification of a critical exon common to all transcript variants that when deleted disrupts gene function (Skarnes PMID: 21 677 750). For the *Baiap2l2* gene, a promoter‐driven targeting cassette was used to generate a ‘knockout‐first’ allele (tm1a) in C57BL/6N embryonic stem cells. Insertion of the *lacZ* trapping cassette and a floxed promoter‐driven *neo* cassette inserted into intron 3 of the gene is expected to disrupt gene function. Cre‐mediated deletion of the selection cassette and floxed exon 4 of the *tm1a* allele generates a *lacZ*‐tagged allele (*tm1b*), which was used for the present study. Abbreviations: *FRT*, flippase recognition target; neo, neomycin resistance gene; pA, polyadenylation site; SA, splice acceptor. *B*, generation of the null allele of *Baiap2l2* using CRISPR/Cas9. gRNA was targeted to exon 4, and several mouse lines containing indels were obtained. We chose a line that had a 16 bp deletion (coding sequence deletion: c.247_262delCGGCACTTGAACTCAG; protein truncation: p.Arg83Thrfs^*^67). The protein product is predicted to be truncated in the I‐BAR domain. We referred to the allele we used here as *Baiap2l2^Δ16^*. Mice were backcrossed to C57BL/6 for more than six generations to minimize any off‐target modifications.

*Baiap2l2*‐deficient mice (*Baiap2l2^tm1b^*) were produced through Cre‐mediated conversion of the ‘knockout‐first’ tm1a allele, which was achieved by treating IVF derived embryos with a cell permeable Cre‐enzyme (Fig. 1 *A*). In the converted tm1b allele, exon 4 (ENSMUSE00000126561) of the *Baiap2l2* gene (ENSMUSG00000018126; MGI:2 652 819), located on chromosome 15, is deleted, leaving a lacZ reporter cassette. Exon 4 encodes part of the I‐BAR domain, which is critical for BAIAP2L2 function, and the *lacZ* cassette contains a splice acceptor that subsumes normal splicing (Fig. 1 *A*). We also used CRISPR/Cas9 gene editing to generate a mouse line (*Baiap2l2^Δ16^*) with a 16 nucleotide deletion in exon 4 (c.247_262delCGGCACTTGAACTCAG p.Arg83Thrfs^*^67) (Fig. 1 *B*). Both transgenic mouse lines (UK: *Baiap2l2^tm1b^*; USA: *Baiap2l2^Δ16^*) showed the same morphological and physiological phenotypes.

### *Baiap2l2^tm1b/tm1b^* mice exhibit early progressive hearing loss

**Figure 2:**
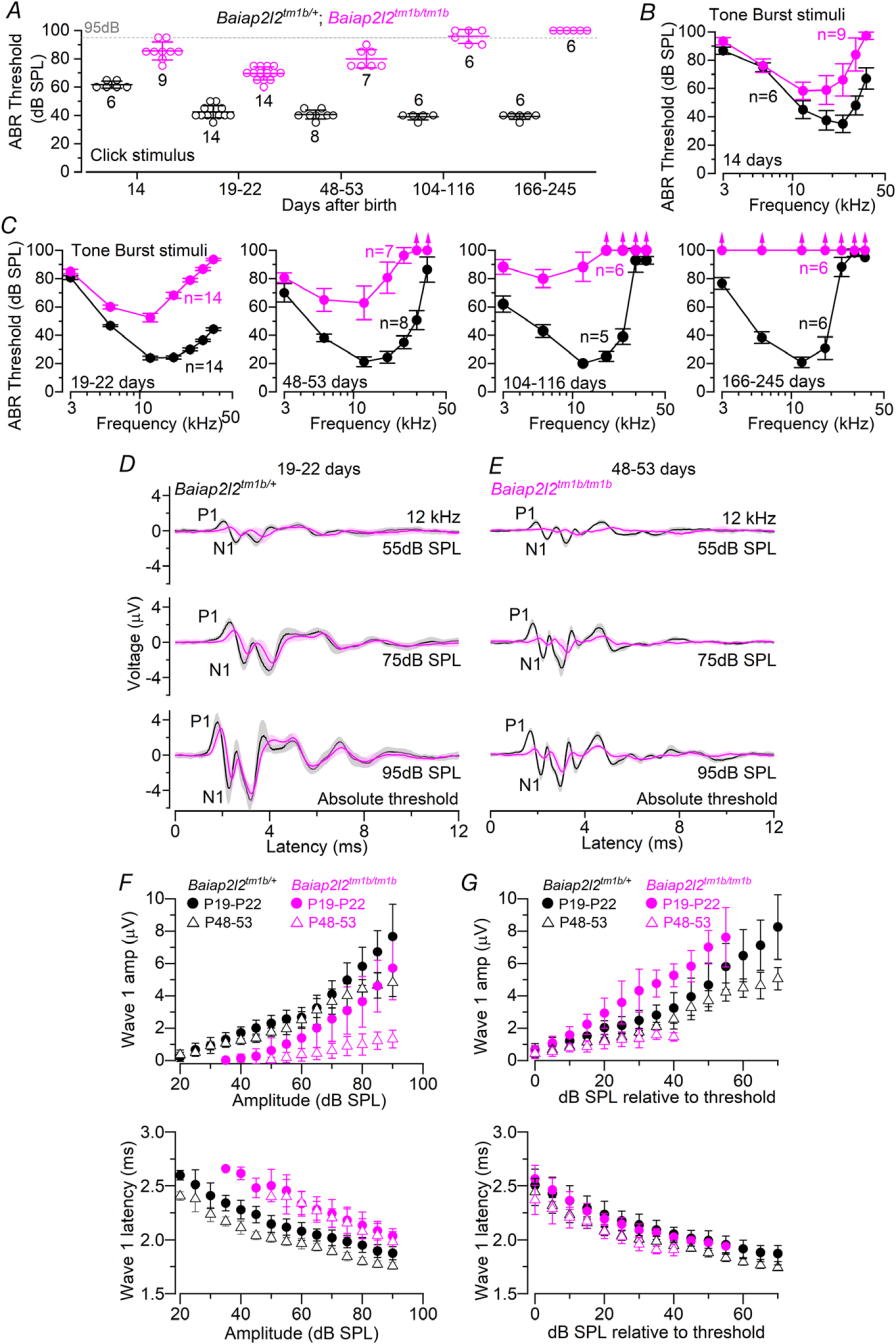
ABR thresholds evoked in *Baiap2l2 *mice *A*, average ABR thresholds for click stimuli recorded from *Baiap2l2* control (*Baiap2l2^tm1b/+^*) and knockout littermate mice (*Baiap2l2^tm1b/tm1b^*) of increasing ages. The dashed line represents the upper threshold limit of our system, 95dB. The number of mice tested is shown above or below the average data points (closed symbols) and single data points are plotted as small open symbols. *B* and *C*, ABR thresholds for frequency‐specific pure tone stimulation from 3 kHz to 36 kHz recorded from *Baiap2l2^tm1b/+^*and *Baiap2l2^tm1b/tm1b^* littermate mice at 14 days (*B*) and 19–22 days, 48–53 days, 104–116 days and 166–245 days (*C*) after birth. The number of mice tested for each age/strain is shown. *D* and *E*, average ABR waveform responses at 12 kHz at increasing stimulus intensity (dB SPL) relative to threshold at postnatal day 19–22 (*Baiap2l2^tm1b/+^*: *n* = 14; *Baiap2l2^tm1b/tm1b^*: *n* = 14) and 48–53 (*Baiap2l2^tm1b/+^*: *n* = 8; *Baiap2l2^tm1b/tm1b^*: *n* = 7). Continuous lines represent the average values and the shaded areas the SD. P1 and N1 indicate the positive and negative peaks of wave 1, respectively. *F* and *G*, average amplitude (from P1 to N1: top) and latency of wave 1 (time between the onset of the stimulus and P1: bottom) as a function of dB SPL (*F*) and relative to threshold (*G*) in both strains at 19–22 days and 48–53 days. In (*F*), the wave 1 amplitude of ABR signals below thresholds were set to zero, and were not used to measure the latency. For the individual recordings used to calculate the averages shown in (*B*), (*C*), (*F*) and (*G*), see Supporting information, Data S1.

The hearing sensitivity of *Baiap2l2^tm1b^* mice was tested using ABRs. Control mice (*Baiap2l2^tm1b/+^*) had normal thresholds for clicks at P14 that decreased to even lower sound pressure levels over the first post‐hearing week (*P < *0.0001, Tukey's post test, one‐way ANOVA), as previously shown in wild‐type mice (Song *et al*. 2006), and then remained constant up to at least 245 days of age (∼8 months, *P = *0.1357, one‐way ANOVA) (Fig. 2 *A*). *Baiap2l2* knockout littermate mice (*Baiap2l2^tm1b/tm1b^*) exhibited a similar initial decrease in click ABR thresholds (*P < *0.0001, Tukey's post test), but, by 2 months of age, thresholds were elevated back to levels seen at 14 postnatal days (*P = *0.2185, Tukey's post test). Despite the comparable initial trend, ABR thresholds for clicks were significantly elevated in *Baiap2l2^tm1b/tm1b^* compared to control mice (*P* < 0.0001, two‐way ANOVA) (Fig. 2 *A*). By 5–8 months of age (166–245 days) (Fig. 2 *A*), click ABRs were no longer detected in *Baiap2l2^tm1b/tm1b^* mice. Pure‐tone evoked ABRs (3, 6, 12, 18, 24, 30 and 36 kHz) were also found to be significantly elevated at all age‐group tested in *Baiap2l2^tm1b/tm1b^* compared to littermate controls (*P < *0.0001 for all ages: two‐way ANOVA) (Fig. 2 *B* and *C*).

We then analysed the amplitude and latency of the ABR wave 1 at 12 kHz (Fig. 2 *D* and *E*). This frequency was selected because it allows the correlation between ABR data and the *in vitro* results, which were performed in the frequency range 9–12 kHz (see below). We found that the amplitude of wave 1 over the overlapping range was significantly reduced in *Baiap2l2^tm1b/tm1b^* compared to control mice at both age ranges (range: 50–95 dB SPL, *P < *0.0001, Tukey's post test, two‐way ANOVA) (Fig. 2 *F*, upper). Wave 1 latency was significantly increased between the two genotypes at both ages (*P* < 0.0001) (Fig. 2 *F*, lower). When the latency of wave 1 was plotted as a function of the dB relative to threshold, it was no longer significantly increased in *Baiap2l2^tm1b/tm1b^* compared to control mice (range: 0–40 dB SPL, *P* = 0.9677, two‐way ANOVA) (Fig. 2 *G*, lower). By contrast, wave 1 amplitude was still significantly different between the two genotypes at both ages (19–22 days: *P* < 0.0001, 48–53 days: *P* = 0.0468, Tukey's post test, two‐way ANOVA) (Fig. 2 *G*, upper). Because ABRs wave 1 is generated by the summed response to sound of the afferent nerve fibres innervating the IHCs (Møller & Jannetta, 1982; Schaette & McAlpine, 2011), these results suggested that the loss of BAIAP2L2 probably affects the sensory hair cells. Moreover, the increased ABR thresholds and the steeper growth of the wave 1 amplitude in the young adult *Baiap2l2^tm1b/tm1b^* mice (P19‐P22) (Fig. 2 *G*) suggest a possible loss of cochlear compression with increasing stimulus levels, which suggests OHC dysfunction.

**Figure 3:**
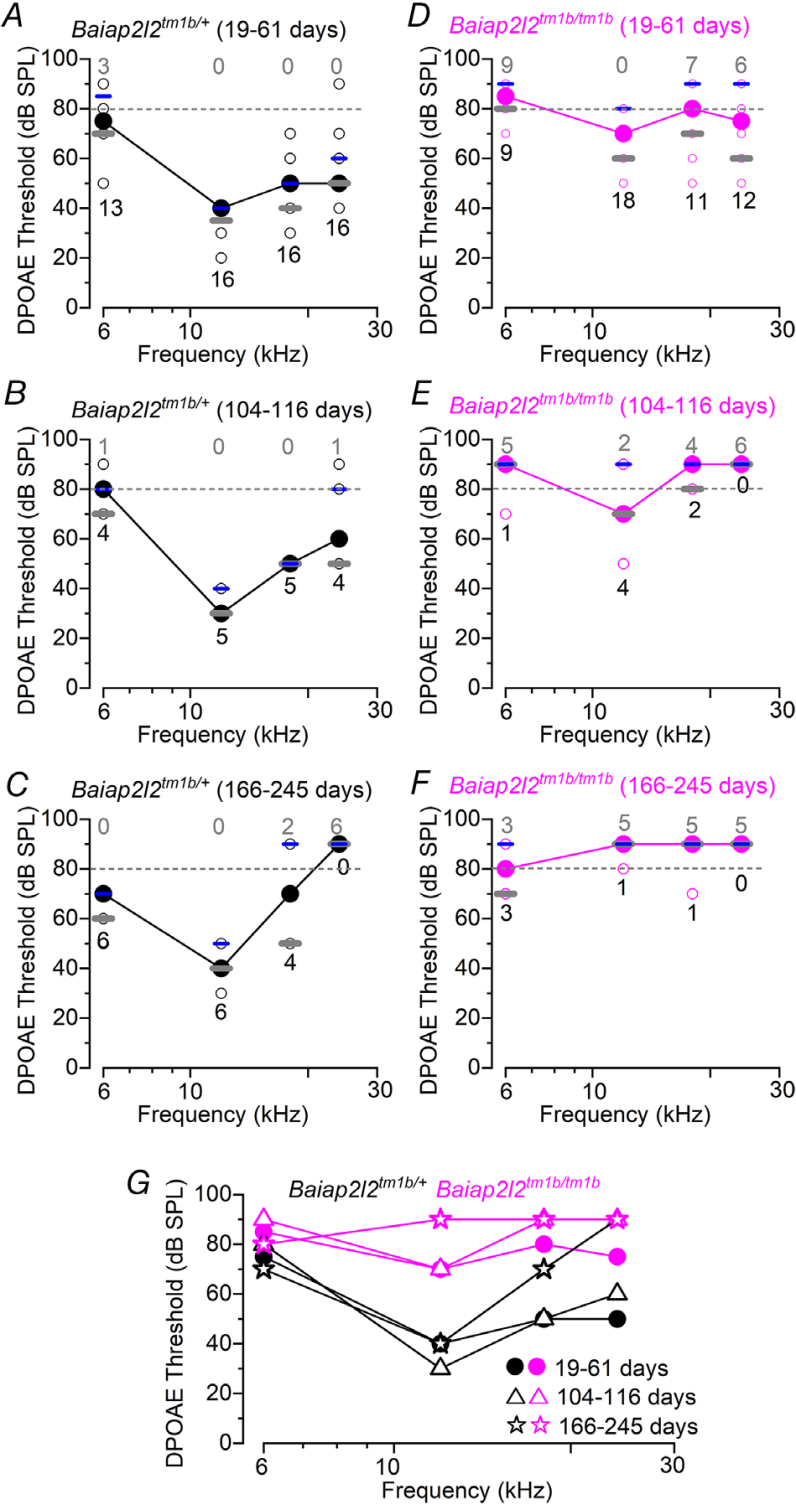
DPOAE thresholds are elevated in *Baiap2l2* knockout mice *A*–*F*, DPOAE thresholds measured from *Baiap2l2^tm1b/+^* (*A–C*) and *Baiap2l2^tm1b/tm1b^* mice (*D–F*) at 19–61 days (*A* and *D*), 104–116 days (*B* and *E*) and 166–145 days (*C* and *F*) after birth. The frequency range tested: 6 kHz, 12 kHz, 18 kHz and 24 kHz. Due to the presence of ‘not‐found’ values (i.e. above the upper threshold limit of our system, 80 dB: dashed lines), values are plotted as the median (line and circles) together with the first (grey short lines) and third (blue short lines) quartiles. Single values are reported as open circles. For each animal, the number of ‘found’ and ‘not‐found’ values at each frequency is shown below and above the median, respectively. *G*, comparison of the median DPOAE thresholds from (*A*) *to* (*F*).

To provide a specific readout of OHC function *in vivo*, we recorded DPOAEs (Fig. 3). DPOAEs are a product of cochlear amplification caused by the displacement of OHC stereociliary bundles during sound stimulation. We found that the DPOAE thresholds over the age range investigated in *Baiap2l2^tm1b/tm1b^* mice were significantly raised compared to littermate controls (*P *< 0.0001 for all three age groups: non‐parametric aligned ranks transformation two‐way ANOVA), indicating that OHCs are probably dysfunctional.

### BAIAP2L2 is localized at the tips of the shorter transducing stereocilia

**Figure 4:**
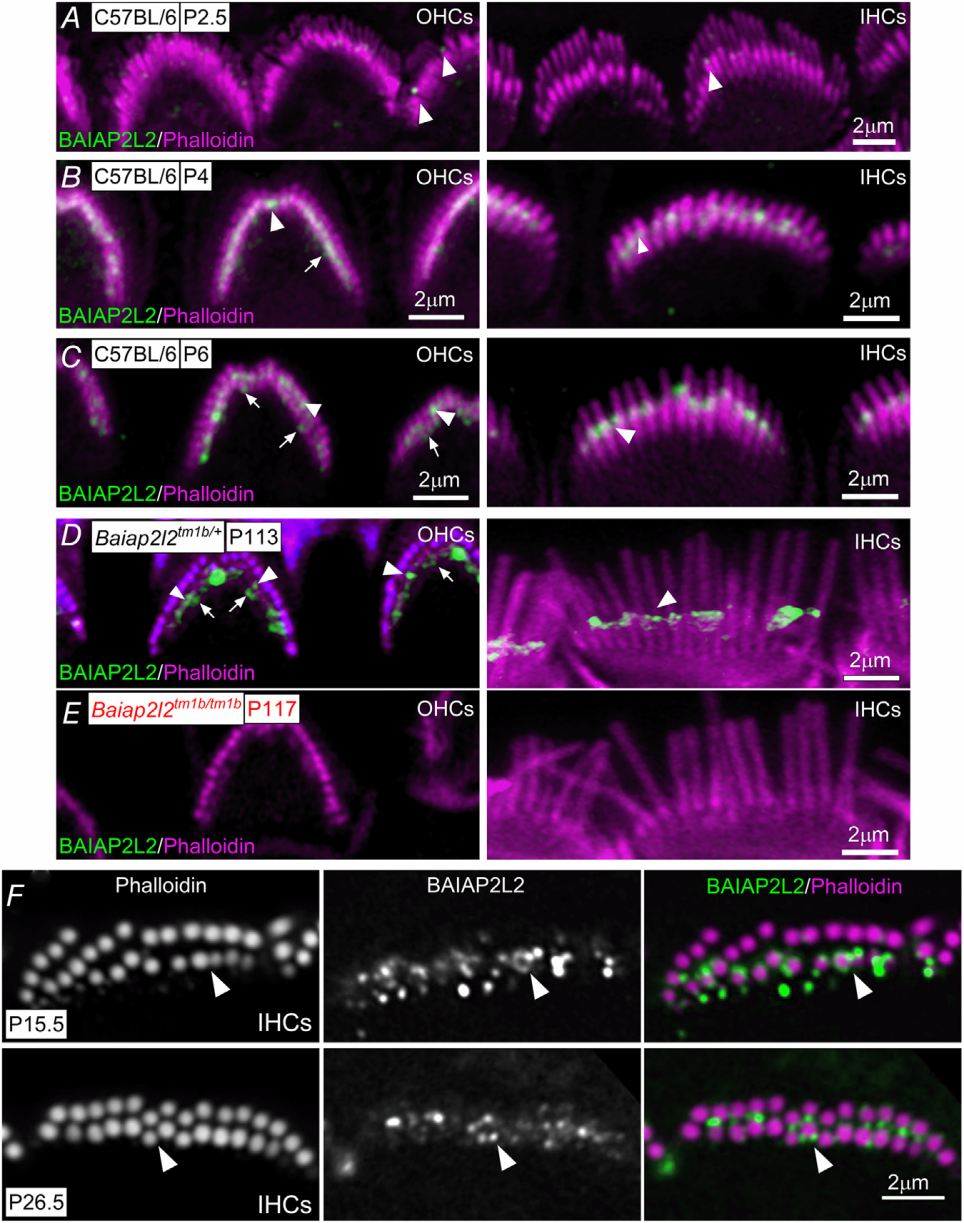
BAIAP2L2 localizes to stereocilia tips *A–D*, antibody against BAIAP2L2 (green) labels its localization at stereocilia tips in apical‐coil OHCs (*A*–*D*, left) and IHCs (*A*–*D*, right) from P2.5 (*A*), P4 (*B*), P6 (*C*) and P113 (*D*) mice. Anti‐BAIAP2L2 was: Atlas Antibodies (HPA003043). Phalloidin (red) labels actin, marking the stereocilia. Note that BAIAP2L2 is localized at the tips of the second (arrowheads) and third (arrows) rows of stereocilia in OHCs, but primarily in the second rows of stereocilia in IHCs (arrowheads). *E*, BAIAP2L2 staining was absent in the stereocilia of OHCs (left) and IHCs (right) from P117 *Baiap2l2^tm1b/tm1b^* mice using the same antibody listed above. *F*, top view of the stereocilia from P15.5 (top) and P26.5 (bottom) IHCs to show that BAIAP2L2 (green) localization shifts to stereocilia shafts in older animals. Single slices shown at approximately mid‐way down the length of row, with instances of clear annular signal indicated by arrowheads.

RNA‐sequencing data have recently shown that in the mouse cochlea *Baiap2l2* is specifically expressed in hair cells within the organ of Corti (https://umgear.org: Kolla *et al*. 2020). The exact localization of BAIAP2L2 in the hair cell was investigated using immunofluorescence labelling with two different antibodies (see Methods). BAIAP2L2 expression was already detectable as early as P2.5 (Fig. 4 *A*) and persisted in the adult cochlea in both hair cell types (Fig. 4 *B* and *D*), although it was absent in hair cells from *Baiap2l2^tm1b/tm1b^* (Fig. 4 *E*) and *Baiap2l2^Δ16/Δ16 ^*mice (data not shown). Within the OHCs, BAIAP2L2 was present in both the second and third rows of stereocilia (Fig. 4 *A*), which correspond to the transducing stereocilia (Beurg *et al*. 2009), whereas, in IHCs, it appeared to localize primarily at the tips of the second stereociliary row (Fig. 4 *B* and *D*). When detected with the abcam antibody, BAIAP2L2 appeared to shift in IHCs during development; while initially concentrated at row 2 tips, BAIAP2L2 localization gradually became more annular and was found along row 2 stereocilia shafts from ∼P14 onwards (Fig. 4 *F*). The modest differences in apparent localization of BAIAP2L2 in older animals may reflect differential exposure of the distinct antibody epitopes.

**Figure 5:**
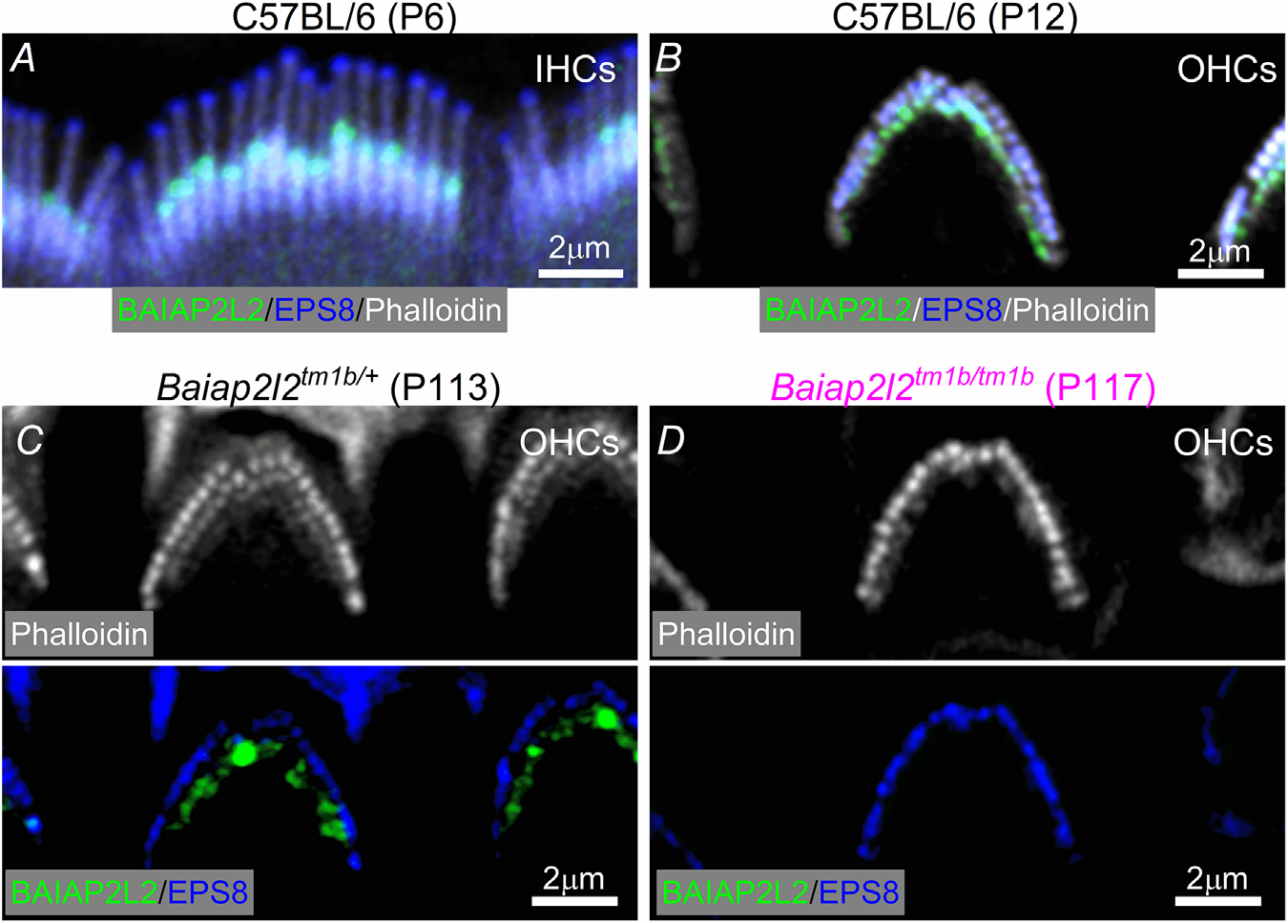
Separate localization of BAIAP2L2 and EPS8 at the stereocilia tips *A–C*, BAIAP2L2 (green) and EPS8 (blue) appear spatially segregated, with the latter primarily located at the tips of the taller rows of stereocilia in P6 IHCs (*A*) and P12 OHCs (*B*) from C57BL/6 mice and P113 OHCs (*C*) from *Baiap2l2^tm1b/+^*. *D*, OHCs from *Baiap2l2^tm1b/tm1b^* mice only showed EPS8 staining. In (*C*) and (*D*), the phalloidin staining (white: stereociliary marker) is shown separately to better visualize the segregated distribution of BAIAP2L2 and EPS8.

BAIAP2L2 localization was different to that of EPS8, which mainly localizes at the tallest row of stereocilia in both young P6‐P12 (Fig. 5 *A* and *B*) and adult P113‐117 mice (Fig. 5 *C* and *D*). By contrast, BAIAP2L2 had a similar localization to that of EPS8L2; both were found at the tips of rows 2 and 3, the shorter transducing stereocilia (Furness *et al*. 2013).

### Progressive loss of the transducing rows of stereocilia in *Baiap2l2* knockout mice

**Figure 6:**
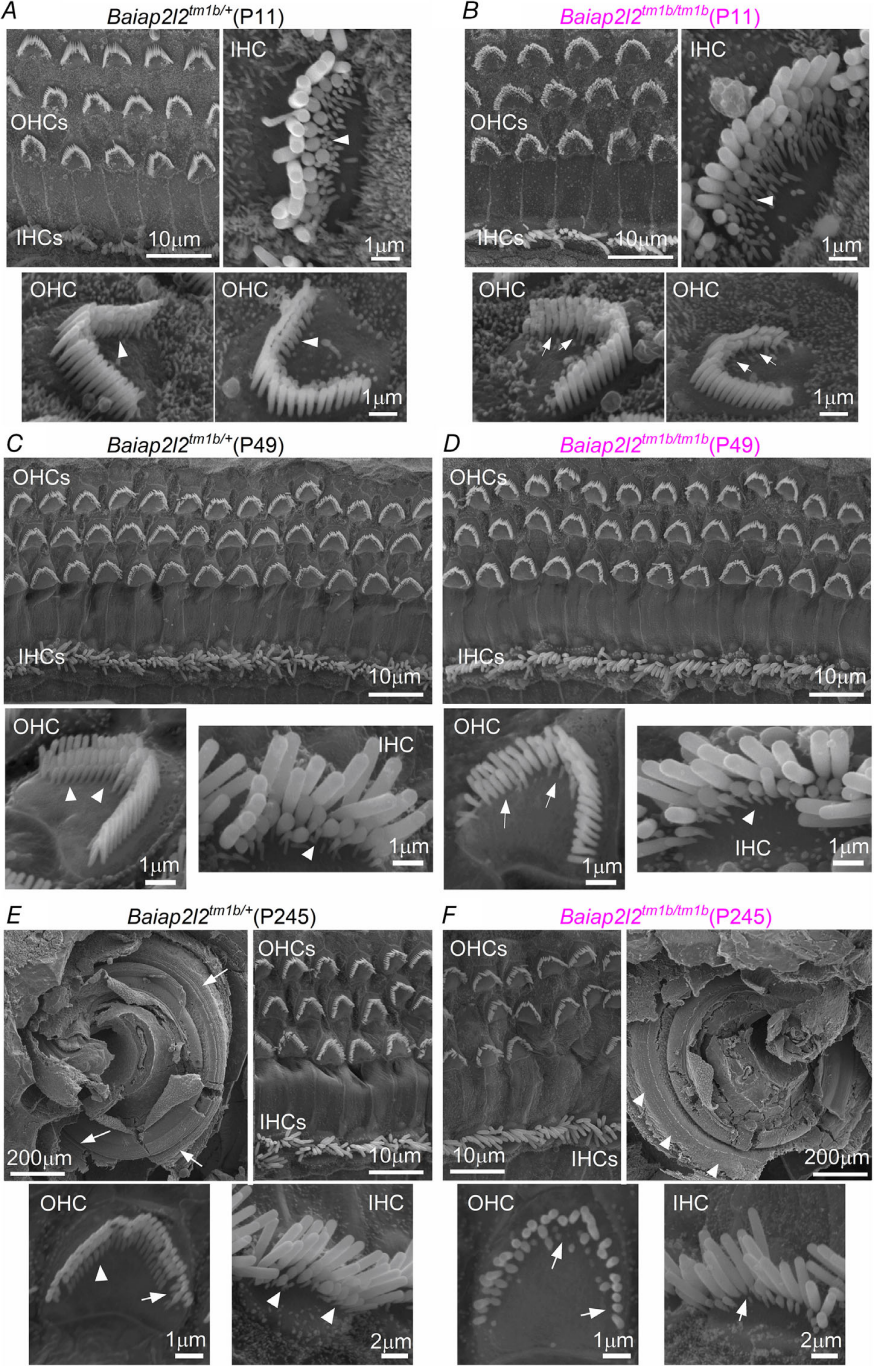
Hair bundle morphology in hair cells from adult *Baiap2l2 *mice *A–F*, scanning electron micrographs showing the typical hair bundle structure from apical‐coil OHCs and IHCs in *Baiap2l2^tm1b/+^* (*A*, *C* and *E*) and *Baiap2l2^tm1b/tm1b^* mice (*B*, *D* and *F*) at pre‐hearing stages of development (P11: *A* and *B*), at P49 (*C* and *D*) and at P245 (*E* and *F*). Note that, generally, hair bundles are composed of three rows of stereocilia: tall, intermediate and short. Arrowheads indicate the presence of the third row of stereocilia; arrows indicate missing stereocilia in *Baiap2l2* knockout OHCs. *E* and *F*, also shows a low magnification SEM illustrating the gross morphology of the apical coil portion of the cochlea control (*E*, top left) and *Baiap2l2^tm1b/tm1b^* (*F*, top right) mice at P245 days of age. Note that the hair bundles of the hair cells are gradually disappearing from the surface of the epithelium in *Baiap2l2^tm1b/tm1b^* (arrowheads in *F*) but still present in littermate controls (arrows in *E*).

**Figure 7:**
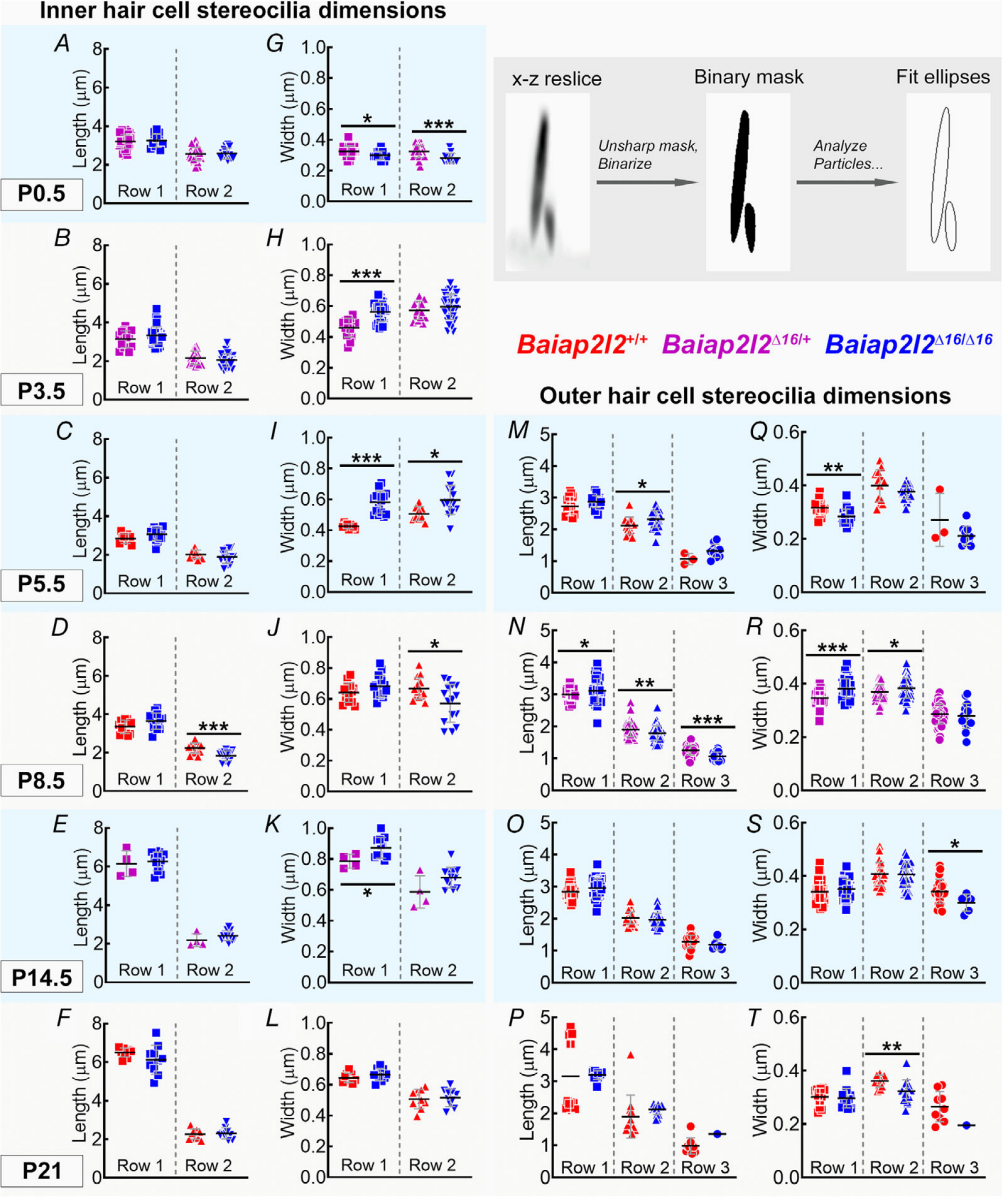
Bundle morphology is normal at early stages of development in both IHCs and OHCs *A–L*, IHC stereocilia lengths and widths for rows 1 and 2. Row 3 stereocilia of IHCs are typically not resolvable by fluorescence, and thus are not measured here. P0.5 from *Baiap2l2^Δ16/+^* (magenta: *n* = 42 stereocilia profiles from 15 IHCs, 4 cochleae), *Baiap2l2^Δ16/Δ16^* (blue: *n* = 12 stereocilia profiles from 5 IHCs, 2 cochleae). P3.5 *Baiap2l2^Δ16/+^* (*n* = 15 stereocilia, 5 IHCs, 3 cochleae), *Baiap2l2^Δ16/Δ16^* (*n* = 42 stereocilia, 15 IHCs, 6 cochleae). P5.5 *Baiap2l2^+/+^* (red, *n* = 9 stereocilia, 5 IHCs, 2 cochleae), *Baiap2l2^Δ16/Δ16^* (*n* = 16 stereocilia, 7 IHCs, 2 cochleae). P8.5 *Baiap2l2^+/+^* (*n* = 13 stereocilia, 5 IHCs, 1 cochlea), *Baiap2l2^Δ16/Δ16^* (*n* = 15 stereocilia, 6 IHCs, 2 cochleae). P14.5 *Baiap2l2^Δ16/+^* (*n* = 4 stereocilia, 3 IHCs, 1 cochlea), *Baiap2l2^Δ16/Δ16^* (*n* = 12 stereocilia, 6 IHCs, 1 cochlea). P21 *Baiap2l2^+/+^* (*n* = 9 stereocilia, 3 IHCs, 1 cochlea), *Baiap2l2^Δ16/Δ16^* (*n* = 10 stereocilia, 7 IHCs, 3 cochleae). *M–T*, OHC stereocilia lengths and widths for rows 1, 2 and 3. The number of measurable row 3 stereocilia decreases by P21. P5.5 *Baiap2l2^+/+^* (*n* = 13 stereocilia, 6 OHCs, 2 cochleae), *Baiap2l2^Δ16/Δ16^* (*n* = 23 stereocilia, 5 OHCs, 1 cochleae). P8.5 *Baiap2l2^Δ16/+^* (*n* = 73 stereocilia, 15 OHCs, 4 cochleae), *Baiap2l2^Δ16/Δ16^* (*n* = 45 stereocilia, 9 OHCs, 2 cochleae). P14.5 *Baiap2l2^+/+^* (*n* = 31 stereocilia, 7 OHCs, 3 cochleae), *Baiap2l2^Δ16/Δ16^* (*n* = 38 stereocilia, 9 OHCs, 3 cochleae). P21 *Baiap2l2^+/+^* (*n* = 12 stereocilia, 5 OHCs, 2 cochleae), *Baiap2l2^Δ16/Δ16^* (*n* = 15 stereocilia, 8 OHCs, 2 cochleae). All measurements were made from apical turn of the cochlea. Pairwise comparisons between genotypes used *t* tests (two‐tailed, assumption of equal variance). Individual measurements of each stereocilia are plotted separately and overlaid with their mean ± SD. The inset shows an example stereocilia pair from P21 IHCs of *Baiap2l2^+/+^* mice, demonstrating extraction of length and width measurements from fit ellipses applied to a processed *x*–*z* reslice (panel width of 4 μm).

Because BAIAP2L2 is expressed in stereociliary bundles, we investigated whether its absence caused defects in the normal growth and/or maintenance of the stereocilia in both OHCs and IHCs. Using SEM, we found that, at P11, the hair bundles of *Baiap2l2^tm1b/tm1b^* mice showed a few missing stereocilia in the shorter third row in OHCs, but not in IHCs (Fig. 6 *A* and *B*). A similar phenotype has previously been described in *Eps8l2* knockout mice, although only in the mature cochlea (Furness *et al*. 2013). At P49, the third row of stereocilia in OHCs was almost completely missing in the *Baiap2l2^tm1b/tm1b^* mice, whereas IHCs appeared to have a normal complement of stereocilia (Fig. 6 *C* and *D*). By P245, a time when *Baiap2l2^tm1b/tm1b^* mice are completely deaf (Fig. 2), the stereocilia of both OHCs and IHCs lacking BAIAP2L2 were almost exclusively formed by one row of stereocilia and several hair cells were missing in the sensory epithelium (Fig. 6 *E* and *F*). These data indicate that the progression of the stereocilia defects as a result of the absence of BAIAP2L2 was different between OHCs and IHCs, with the latter being delayed by several weeks. We quantified stereocilia dimensions of P0.5 to P21 IHCs and of P5.5 to P21 OHCs from *Baiap2l2^Δ16 ^*mice by measuring the apparent length and width of the phalloidin‐stained actin cores (Krey *et al*. 2020). Although pairwise comparisons for row dimensions between genotypes at each age yielded several significant differences, no consistent trend in the direction of the differences was observed for either IHCs (Fig. 7 *A–L*) or OHCs (Fig. 7 *M–T*). We also noted the loss of row 3 stereocilia from P21 *Baiap2l2^Δ16/Δ16 ^*mice (Fig. 7 *P* and *T*), which is in agreement with the above SEM data (Fig. 6).

### MET currents are reduced in *Baiap2l2* knockout mice

**Figure 8:**
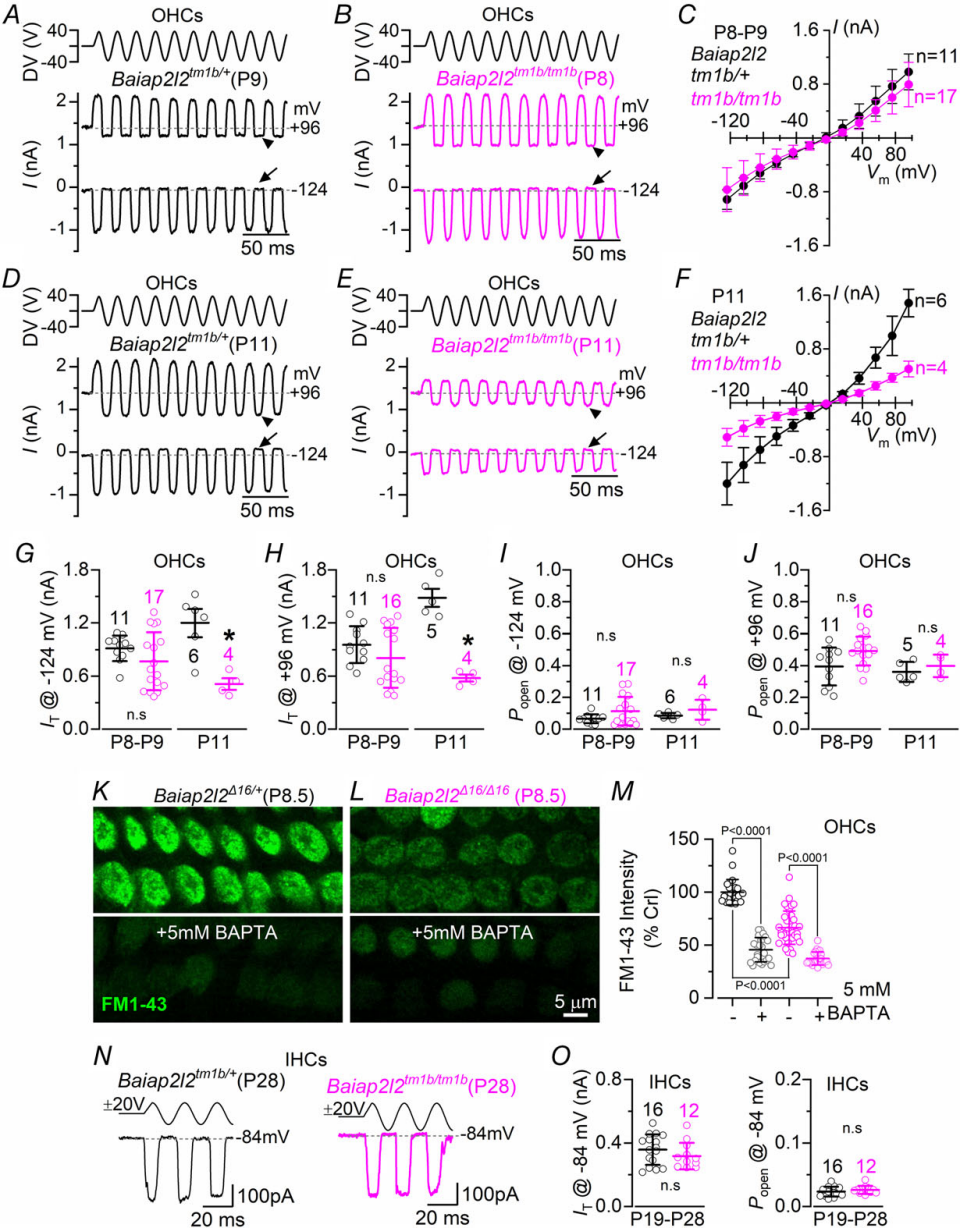
Mechanoelectrical transduction is reduced in *Baiap2l2^tmtb^* mice *A* and *B*, saturating MET currents in apical OHCs from control *Baiap2l2^tm1b/+^* (*A*, P9) and *Baiap2l2^tm1b/tm1b^* (*B*, P8) mice in response to 50 Hz sinusoidal force stimuli to the hair bundles at membrane potentials of −124 and +96 mV. Driver voltage (DV) stimuli to the fluid jet are shown above the traces, with positive deflections of the DV being excitatory. The arrows and arrowheads indicate the closure of the transducer channel in response to inhibitory bundle stimuli at −124 and +96 mV, respectively. *C*, average peak to peak MET current–voltage curves from apical OHCs of control *Baiap2l2^tm1b/+^* (*n* = 11) and littermate *Baiap2l2^tm1b/tm1b^* (*n* = 17) mice. Recordings were obtained by mechanically stimulating the hair bundles of OHCs at the same time as stepping their membrane potential from −124 mV to +96 mV in 20 mV increments. *D–F*, saturating MET current recorded in P11 OHCs from control (*n* = 6) and littermate *Baiap2l2^tm1b/tm1b^* (*n* = 4) mice using the same protocols described in (*A*) to (*C*). For the single‐data recordings used to calculate the averages shown in (*C*) and (*F*), see Supporting information, Data S1. *G* and *H*, maximal size of the MET current in both genotypes measured at −124 mV (*G*) and +96 mV (*H*) at the two age ranges investigated in (*A*) to (*F*). *I* and *J*, resting open probability (*P*_o_) of the MET current in OHCs from the two genotypes and at the two ages tested from the holding of −124 mV (*I*) and +96 mV (*J*). The resting current is given by the holding current minus the current present during inhibitory bundle deflection. The *P*_o_ was found not to be significantly different between the two genotypes, even though the size of the MET current was largely reduced at P11 (−124 mV: *P = *0.1950; +96 mV: *P = *0.4225, *t* test). At P8–P9, *P_o_* was also not significantly different between the two genotypes (−124 mV: *P = *0.1035; +96 mV: *P = *0.0680). For average data values, see Supporting information, Statistical Summary Document. *K*–*M*, example experiment for FM1‐43 uptake by OHCs from P8.5 *Baiap2l2^Δ16/+^* (*K*) and P8.5 *Baiap2l2^Δ16/Δ16 ^*mice with and without the application of 5 mm BAPTA, with quantification (*L*). Representative results shown from a single experiment (*n* = 24–36 OHCs per condition). The statistical test was performed via one‐way ANOVA, Sidak's multiple comparison test. *N*, saturating MET currents in apical IHCs from *Baiap2l2^tm1b/+^* (left, P28) and *Baiap2l2^tm1b/tm1b^* (right, P28) mice in response to 50 Hz sinusoidal force stimuli to the hair bundles at membrane potentials of −84. Driver voltage (DV) stimuli to the fluid jet are shown above the traces. *O*, maximal size of the MET current (left) and resting open probability (*P*_o_: right) in adult IHCs from both genotypes measured at −84 mV.

Considering that the stereociliary bundles of *Baiap2l2^tm1b/tm1b^* mice appear to lose the third row of stereocilia prior the onset of hearing (Fig. 6), we investigated possible functional changes in the MET apparatus. MET currents were recorded from P8 to P11 apical coil OHCs (Fig. 8) by displacing their stereociliary bundles using a 50 Hz sinusoidal force stimulus from a piezo‐driven fluid‐jet (Corns *et al*. 2014; Corns *et al*. 2018). Moving the bundles in the excitatory direction (i.e. towards the taller stereocilia) and at negative membrane potentials elicited a large inward MET current in all OHCs tested at P8–P9 (Fig. 8 *A–C*), with a maximal value that was not significantly different between control *Baiap2l2^tm1b/+^* (−915 ± 145 pA, *n* = 11, at −124 mV) and littermate *Baiap2l2^tm1b/tm1b^* mice (−767 ± 327 pA, *n* = 17, *P = *0.1711, *t* test) (Fig. 8 *G*). At P11, as expected during development, the MET currents in control OHCs was significantly larger (−1200 ± 317 pA, *n* = 6) (Fig. 8 *D*, *F* and *G*) compared to that recorded at P8‐P9 (*P = *0.0208). By contrast, the MET current at P11 of *Baiap2l2^tm1b/tm1b^* mice was only about one‐half of that of littermate controls (−511 ± 131 pA, *n* = 4, *P = *0.0036) (Fig. 8 *E–G*). This result is consistent with the loss of row 3 stereocilia and their transducer channels. By stepping the membrane potential from −124 mV to more depolarized values in 20 mV increments, the transducer current decreased in size at first and then reversed near 0 mV in both genotypes (Fig. 8 *C* and *F*), consistent with the non‐selective permeability of MET channels to cations. Note that the current became outward when excitatory bundle stimulation was applied during voltage steps positive to the reversal potential of the transducer current. The maximal MET current at +96 mV (Fig. 8 *H*) was also not significantly different between the two genotypes at P8‐P9 (*Baiap2l2^tm1b/+^*: +955 ± 206 pA, *n* = 11; *Baiap2l2^tm1b/tm1b^*: +805 ± 337 pA, *n* = 5, *P = *0.2020, *t test*) but highly reduced at P11 (*Baiap2l2^tm1b/+^*: +1484 ± 205 pA, *n* = 17; *Baiap2l2^tm1b/tm1b^*: +579 ± 79 pA, *n* = 4, *P < *0.0001).

The resting MET current, which is the current flowing through open transducer channels in the absence of mechanical stimulation, can be measured from the difference between the holding current and the current present during inhibitory bundle deflection. Experimentally, this inhibitory stimulation is obtained when negative driver voltages pull solution into the fluid jet and completely closes the MET channel. At negative membrane potentials, the resting current was evident in OHCs from both control (Fig. 8 *A* and *D*, arrowheads) and *Baiap2l2^tm1b/tm1b^* mice (Fig. 8 *B* and *E*, arrowheads). At positive potentials, the larger resting transducer current (Fig. 8 *A*, *B*, *D* and *E*, arrows) is the result of an increased open probability of the transducer channel consequential to a reduced driving force for Ca^2+^ influx (Crawford *et al*. 1989; Corns *et al*. 2014). The resting MET current was represented as a proportion of the total MET current (i.e. resting open probability, *P*_o_) and was similar between the two genotypes at both negative (Fig. 8 *I*) and positive (Fig. 8 *J*) membrane potentials.

We further tested whether the resting MET current was significantly reduced in adult P8.5 OHCs by using the styryl dye FM1‐43, a permeant blocker of the hair cell MET channel that has previously been used to assess the presence of the resting transducer current (Gale *et al*. 2001). Bath application of FM1‐43 labelled control OHCs, although dye loading was considerably reduced in *Baiap2l2^Δ16/Δ16^* cells (Fig. 8 *K–M*). FM1‐43 uptake in OHCs of *Baiap2l2^Δ16/Δ16 ^*mice was further decreased when the hair bundles were treated with the Ca^2+^ chelator BAPTA (Fig. 8 *M*), which is known to break the tip links (Zhao *et al*. 1996). Considering that the *P_o_* of MET channel in OHCs is not affected (Fig. 8 *I* and *J*), these FM1‐43 data support the suggestion that loss of BAIAP2L2 does not affect the properties of MET channels but rather reduces the number of transducing stereocilia as shown by the SEM data (Fig. 6).

To confirm whether the apparently normal stereociliary bundles of IHCs from young adult *Baiap2l2^tm1b/tm1b^* mice (Fig. 6 *D*) were associated with normal mechanoelectrical transduction, we measured the MET current by displacing their hair bundles as described for OHCs, although only at the holding potential of −84 mV. This more restricted experimental protocol was a result of the nature of the technically demanding MET current recordings from adult IHCs. Moving the bundles of P19–P28 IHCs in the excitatory direction elicited an inward MET current with a maximal size that was not significantly different between *Baiap2l2^tm1b/+^* (−358 ± 96 pA, *n* = 16) and littermate *Baiap2l2^tm1b/tm1b^* mice (−317 ± 84 pA, *n* = 12, *P* = 0.2486, *t* test) (Fig. 8 *N* and *O*). Also, no statistically significant difference was present for the resting open probability between the two genotypes (*P* = 0.3229) (Fig. 8 *O*). These data suggest that the absence of BAIAP2L2 in IHCs, which is expressed in all hair cells from just after birth (Fig. 4), has a late effect on these cells possibly as a result of compensatory mechanisms (see Discussion).

### Basolateral membrane properties develop normally in *Baiap2l2^tm1b/tm1b^* mice

**Figure 9:**
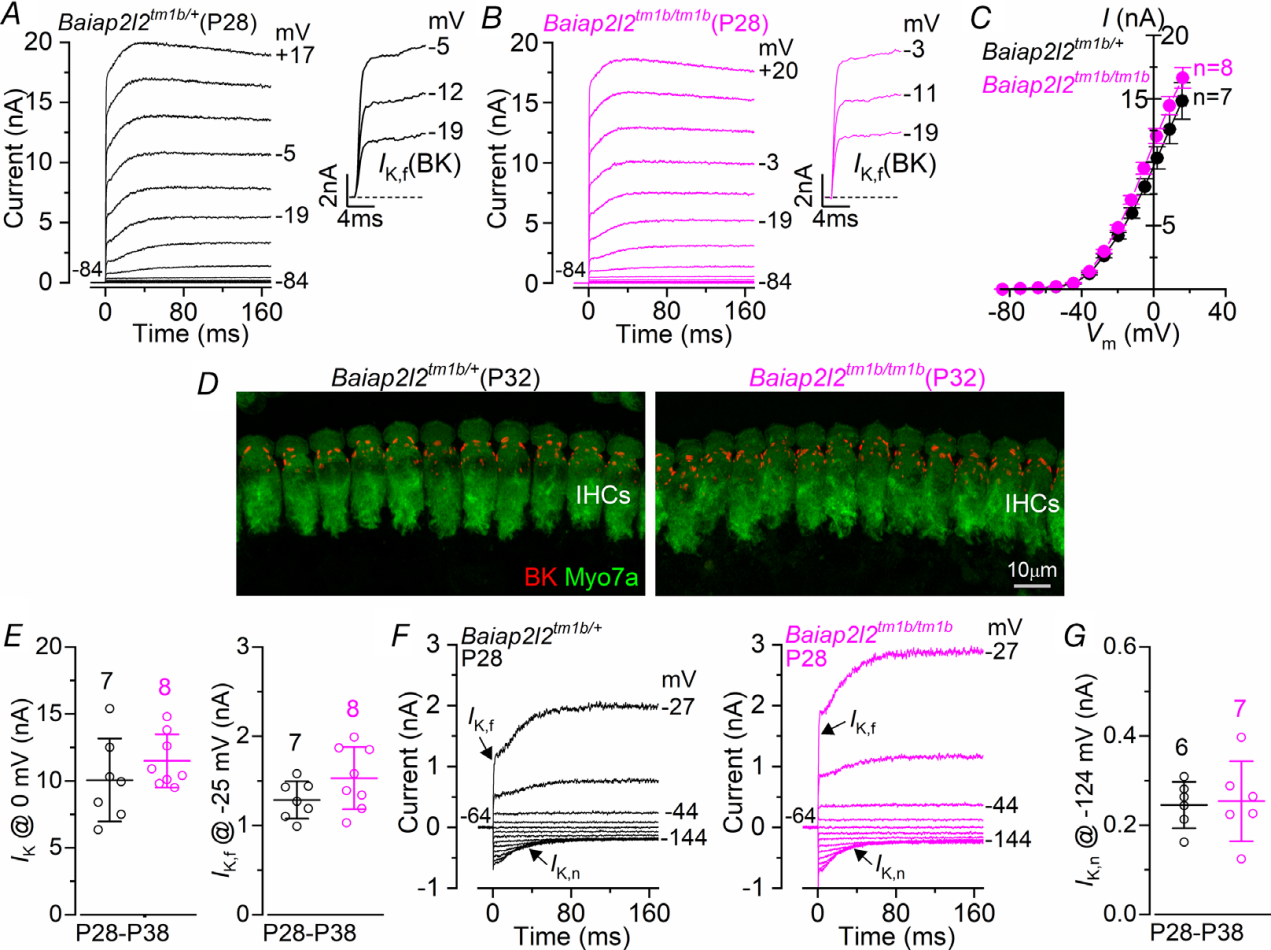
The basolateral membrane properties of adult IHCs are indistinguishable between control and littermate *Baiap2l2^tm1b/tm1b^* mice *A* and *B*, current responses from IHCs of control *Baiap2l2^tm1b/+^* (*A*) and *Baiap2l2^tm1b/tm1b^* (*B*) P28 mice. Current recordings were elicited by using depolarizing voltage steps (10 mV increments) from the holding potential of −84 mV to the various test potentials shown by some of the traces. The fast activation of the BK current (*I*_K,f_) is better appreciated in the expanded time scale (insets). *C*, steady‐state current–voltage curves obtained from IHCs of control (P28‐38) and *Baiap2l2^tm1b/tm1b^* (P28‐35) mice. For the single‐data recordings used to calculate the averages shown in (*C*), see Supporting information, Data S1. *D*, maximum intensity projections of confocal z‐stacks taken from the apical cochlear region of control and *Baiap2l2^tm1b/tm1b^* P32 mice using antibodies against BK (red) and the hair cell marker Myo7a (green). *E*, size of the outward K^+^ current *I*_K,f_, which was measured at −25 mV and at 1 ms from the onset of the voltage step (Marcotti *et al*. 2003). The number of IHCs recorded is shown above each column. *F*, current responses from IHCs of control *Baiap2l2^tm1b/+^* and *Baiap2l2^tm1b/tm1b^* P28 mice, elicited by using hyperpolarizing and depolarizing voltage steps (10 mV increments) from the holding potential of −64 mV to the various test potentials shown by some of the traces. This protocol is used to emphasize the presence of *I*_K,n_. *G*, size of *I*_K,n_, which was measured as the difference between the peak and steady state of the deactivating inward current at –124 mV. Single cell value recordings (open symbols) are plotted behind the average data. The number of IHCs investigated is shown above the average data points. The average IHC resting membrane potential (*V*_m_) and membrane capacitance (*C*_m_) were not significantly different between *Baiap2l2^tm1b/+^* (*V*_m_ −70.4 ± 2.0 mV, *n* = 8; *C*_m_ 11.4 ± 2.4 pF, *n* = 8) and *Baiap2l2^tm1b/tm1b^* mice (*V*_m_ −70.7 ± 3.5 mV, *n* = 9, *P = *0.8275; *C*_m_ 12.2 ± 2.9 pF, *n* = 8, *P* = 0.5606, *t* test).

**Figure 10:**
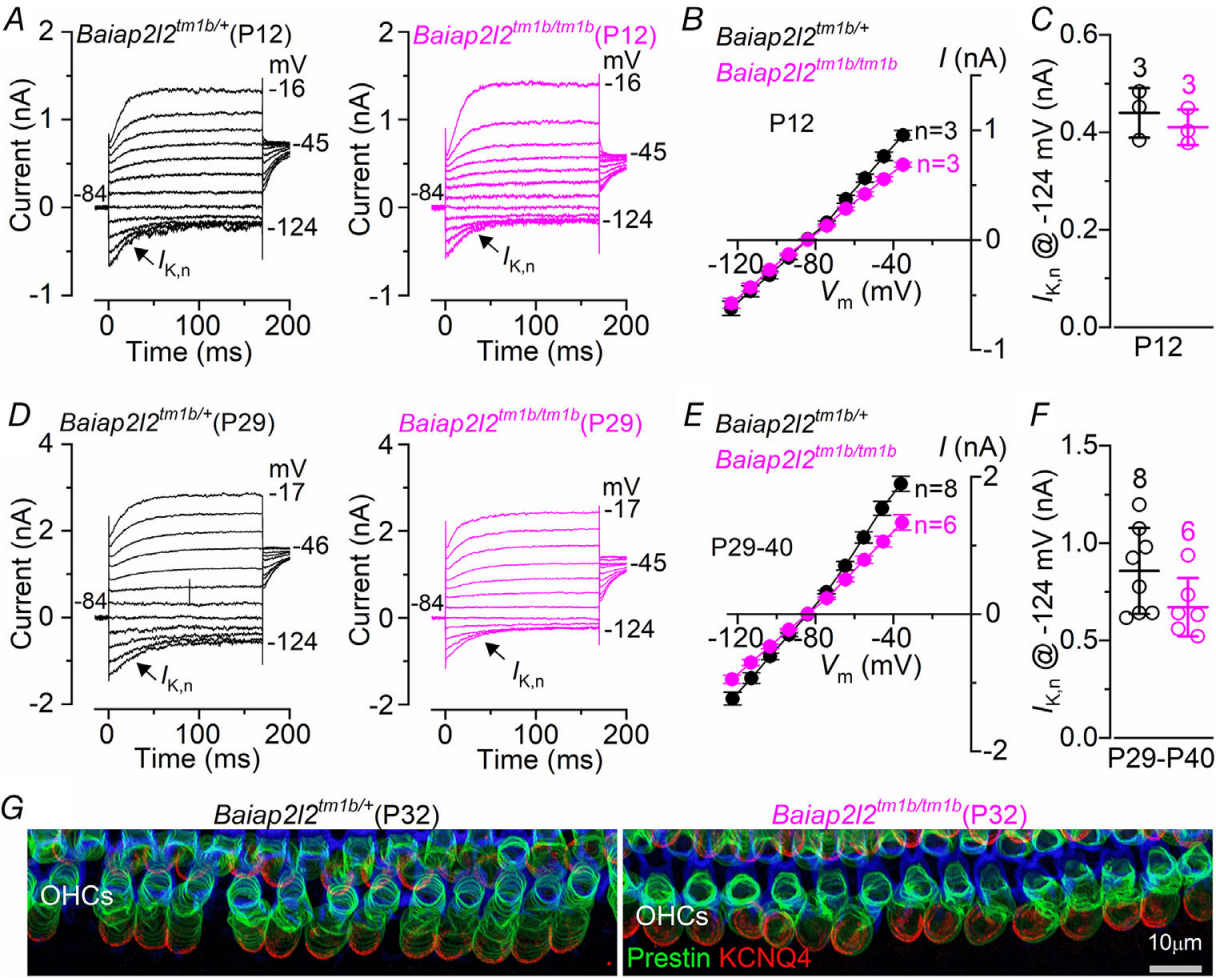
The basolateral membrane properties of adult OHCs are preserved in *Baiap2l2^tm1b^* mice *A–F*, current responses from OHCs of control (*Baiap2l2^tm1b/+^*) and *Baiap2l2^tm1b/tm1b^* mice at the onset of hearing (P12: *A–C*) and adult mice (P29‐40: *D–F*). Currents were elicited by using depolarizing and hyperpolarizing voltage steps (10 mV increments) from the holding potential of −84 mV to the various test potentials shown by some of the traces. Note that the a large *I*_K,n_, which is carried by KCNQ4 channels, is present in OHCs at all ages and in both genotypes. Peak current–voltage curves obtained from OHCs of control and *Baiap2l2^tm1b/tm1b^* mice are shown in (*B*) (P12) and (*E*) (P29‐40). The size of the isolated *I*_K,n_ (Fig. 9 *E*) was not significantly different between the two genotypes (*C*: P12: *P = *0.4592; *F*: P29–40: *P = *0.1022, *t* test). The number of IHCs recorded is shown next to the current–voltage traces (*B* and *E*) or above each column (*C* and *F*). For the single‐data recordings used to calculate the averages shown in (*B*) and (*E*), see Supporting information, Data S1. *G*, maximum intensity projections of confocal z‐stacks taken from the apical cochlear region of control and *Baiap2l2^tm1b/tm1b^* mice at P32 using antibodies against KCNQ4 (red) and prestin (green). Phalloidin (blue) highlights the cuticular plate of the OHCs. Single cell value recordings (open symbols) are also plotted behind the average closed symbols. The number of OHCs investigated is shown above the average data points. The average OHC resting membrane potential (*V*_m_) and membrane capacitance (*C*_m_) were not significantly different between *Baiap2l2^tm1b/+^* (*V*_m_ −73.1 ± 1.6 mV, *n* = 9; *C*_m_ 10.6 ± 0.6 pF, *n* = 8) and *Baiap2l2^tm1b/tm1b^* mice (*V*_m_ −73.7 ± 1.3 mV, *n* = 6, *P = *0.7528; *C*_m_ 10.1 ± 0.3 pF, *n* = 6, *P* = 0.5449, *t* test).

Considering the substantial hearing loss was observed in young‐adult *Baiap2l2^tm1b/tm1b^* mice (Figs 2 and 3), we investigated whether the absence of BAIAP2L2 caused any additional defects in the basolateral membrane properties of both IHCs and OHCs, which could have directly contributed to the hearing phenotype. Mature IHCs expressed a large outward K^+^ current (*I*_K_), which was present in both *Baiap2l2^tm1b/+^* and *Baiap2l2^tm1b/tm1b^* mice using whole‐cell patch clamp recordings (Fig. 9 *A*–*C*). One characteristic K^+^ current of mature IHCs is the rapid activating, large conductance Ca^2+^‐activated K^+^ current carried by BK channels, named *I*_K,f_ (Kros *et al*. 1998; Marcotti *et al*. 2004; Thurm *et al*. 2005; Lingle *et al*. 2019), which was evident in IHCs from both genotypes using both electrophysiology (Fig. 9 *A*–*C*, insets) and immunostaining (Fig. 9 *D*). The size of the total outward *I*_K_ and *I*_K,f_ were not significantly different between the two genotypes (*I*_K_ *: P = *0.3001; *I*_K,f_: *P = *0.1312, *t* test) (Fig. 9 *E*). Another K^+^ current characteristic of mature IHCs is that carried by KCNQ4 channels (*I*_K,n_: Marcotti *et al*. 2003; Oliver *et al*. 2003), which was also similarly expressed in both genotypes (*P = *0.8122, *t* test) (Fig. 9 *F* and *G*). Different from IHCs, the basolateral membrane K^+^ current in adult OHCs is almost exclusively represented by *I*_K,n_ (Fig. 10 *A* and *B*); see also Marcotti & Kros (1999). As for IHCs, the size of *I*_K,n_ was not significantly affected between control and littermate *Baiap2l2^tm1b/tm1b^* mice in both young adult (P12: *P = *0.4592, *t* test) (Fig. 10 *C*) and older mice (P29‐40: *P = *0.1022) (Fig. 10 *D*–*F*). OHCs from adult *Baiap2l2^tm1b/tm1b^* mice also showed normal size and expression profile of the motor protein prestin (Fig. 10 *G*), which is required for driving non‐linear (voltage‐dependent) capacitance, and is the signature of mature electromotility in OHCs (Abe *et al*. 2007; Jeng *et al*. 2020*c*). We also found that the resting membrane potentials (*V*_m_) and cell surface area (membrane capacitance, *C*_m_) was not significantly different in both IHCs and OHCs from control and *Baiap2l2^tm1b/tm1b^* mice (Figs 9 and 10).

These findings indicate that IHCs and OHCs from *Baiap2l2^tm1b/tm1b^* mice are normal and functional in terms of basolateral properties.

### Interaction of BAIAP2L2 with other stereociliary bundle proteins

**Figure 11:**
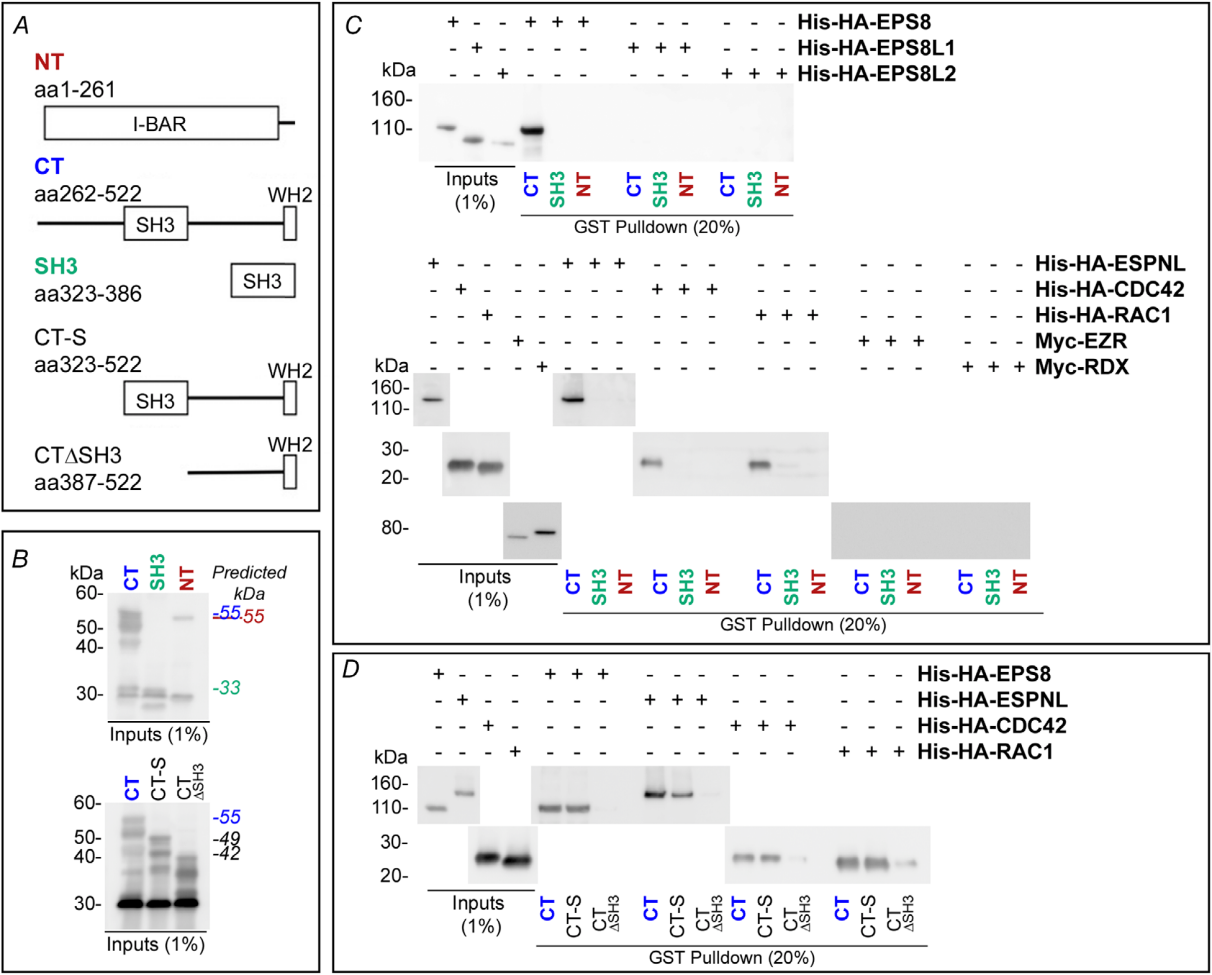
BAIAP2L2 interacts with actin‐associated stereociliary proteins *A*, cartoon diagrams showing the GST‐tagged BAIAP2L2 fusion proteins used for the *in vitro* pull‐down assays (GST tag not included in diagrams). Residue numbering is from the canonical sequence for mouse BAIAP2L2. *B*, example immunoblot of GST inputs used for pull‐down assays. Detected with rabbit anti‐GST. The predicted molecular weight for each of the constructs is indicated on the right, with the text colour corresponding to the text colour for callout above the appropriate lane. *C*, immunoblots with His or Myc inputs and eluates containing BAIAP2L2 complexes from *in vitro* pull‐down assays with *BAIAP2L2* fragments to identify interacting proteins. BAIAP2L2 C‐terminal domain interacted with EPS8, EPSNL, CDC42 and RAC1. Immunoblots detected with mouse anti‐His (EPS8, EPS8L1, EPSL2, ESPNL, CDC42 and RAC1) or rabbit anti‐Myc (EZR and RDX). The volume blotted was 1% for inputs and 20% for pull‐down eluates. *D*, immunoblots with His inputs and eluates containing BAIAP2L2 complexes from *in vitro* pull‐down assays with truncates of the BAIAP2L2 C‐terminal domain to map BAIAP2L2 binding site. The BAIAP2L2 SH3 domain was found to be necessary for the identified interactions. Detected with mouse anti‐His. For (*B*) to (*D*), the volume blotted for input lanes was 1% of the total volume used for the assay, whereas the volume blotted for pull‐down lanes was 20% of the total eluate volume.

We hypothesized that BAIAP2L2 may interact with other proteins known to be concentrated at the tip of the second row of stereocilia, such as EPS8L2 (Furness *et al*. 2013) and ESPNL (Ebrahim *et al*. 2016). We also reasoned that BAIAP2L2 could interact with proteins or protein families known to interact with other I‐BAR members, including the entire EPS8 (Sekerkova *et al*. 2003; Sudhaharan *et al*. 2016) and RHO GTPase (Kast *et al*. 2014; Sudhaharan *et al*. 2016) families. We therefore tested the ability of BAIAP2L2 domains (Fig. 11 *A* and *B*) to interact *in vitro* with a panel of plausible interacting proteins (EPS8, EPS8L1, EPS8L2, ESPNL, CDC42 and RAC1); we also included the membrane‐binding stereocilia proteins EZR and RDX as negative controls. The N‐terminal I‐BAR domain alone served as an additional negative control that was internal to the assay because this domain is considered to primarily interact with membranes (Pykäläinen *et al*. 2011). Recombinant constructs of panel proteins were generated with either an N‐terminal 6xHis tag or an N‐terminal Myc tag, whereas BAIAP2L2 constructs were generated with an N‐terminal GST tag. BAIAP2L2 domain constructs were tested for pull‐down of the panel proteins using the method described in Sekerková *et al*. 2003 with some modifications (see Methods). We found that BAIAP2L2 interacts with CDC42, RAC1, EPS8 and ESPNL, but not with EPS8L1, EPS8L2, EZR or RDX (Fig. 11 *C*). To map where within BAIAPL2L2 these proteins bind, C‐terminal truncations were assayed (Fig. 11 *A* and *B*); the BAIAP2L2 SH3 domain was necessary but not sufficient for the above mentioned interactions to occur (Fig. 11 *D*).

### Dependence of BAIAP2L2 location on stereocilia proteins

**Figure 12:**
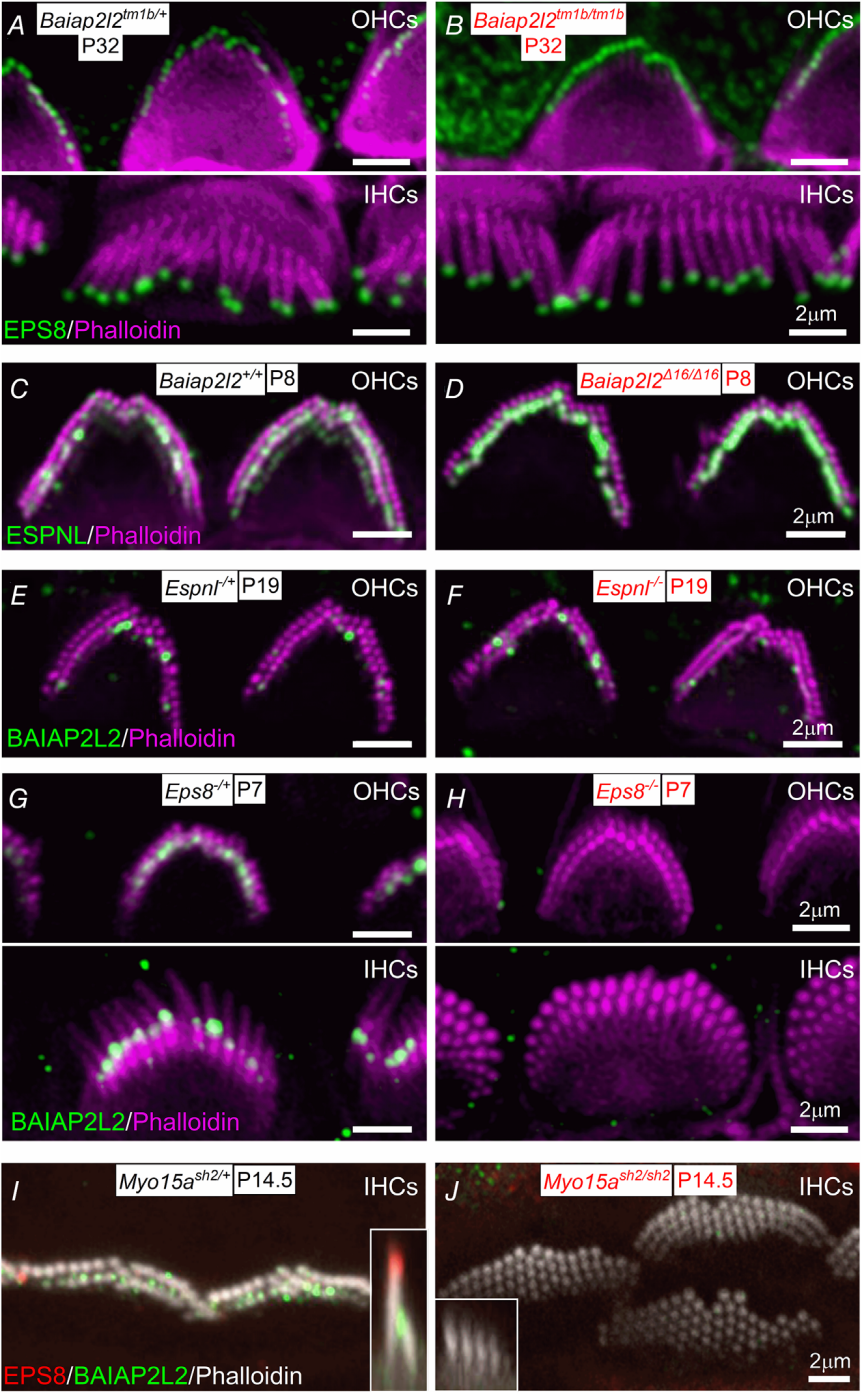
BAIAP2L2 targeting to stereocilia tips requires EPS8 and MYO15A *A* and *B*, EPS8 (green) localization in IHCs and OHCs is normal in *Baiap2l2^tm1b/tm1b^* mice at P32. *C* and *D*, ESPNL is normally localized at the stereocilia of *Baiap2l2^Δ16/Δ16 ^*mice. *E* and *F*, BAIAP2L2 is also present at the stereocilia tip of *Espnl^−/−^* mice. *G* and *H*, BAIAP2L2 does not localize to stereocilia tips of OHCs (upper) and IHC (lower) in *Eps8^−/−^* mice at P7. *I* and *J*, BAIAP2L2 and EPS8 both fail to localize to stereocilia tips in *Myo15a^sh2/sh2 ^*mice. Insets of *x*–*z* reslices to compare stereocilia profiles and respective localization at row 1 and row 2 tips for EPS8 and BAIAP2L2.

We investigated the biological significance of EPS8 and ESPNL interactions with BAIAP2L2 by probing their localization in *Baiap2l2^tm1b/tm1b^* and *Baiap2l2^Δ16/Δ16 ^*mice, and the localization of BAIAP2L2 in *Eps8* and *Espnl* knockouts (Fig. 12 *A–H*). The localization of both EPS8 (Fig. 12 *A* and *B*) and ESPNL (Fig. 12 *C* and *D*) is normal in *Baiap2l2^tm1b/tm1b^* and *Baiap2l2^Δ16/Δ16 ^*mice, respectively; BAIAP2L2 localization in *Espnl* knockout mice is also normal (Fig. 12 *E* and *F*). By contrast, BAIAP2L2 no longer targets to stereocilia tips in *Eps8* knockout mice (Fig. 12 *G* and *H*), which suggests that BAIAP2L2 targeting relies on an interaction with EPS8.

Because EPS8 targeting to the tips of stereocilia requires the motor protein MYO15A (Manor *et al*. 2011; Zampini *et al*. 2011), we tested whether BAIAP2L2 localization also required MYO15A. In mice deficient in MYO15A (*Myo15a^sh2^*: Probst *et al*. 1998), we accordingly found that BAIAP2L2 labelling was absent from stereocilia tips (Fig. 12 *I* and *J*). This result indicates that the transport of BAIAP2L2 is similar to that of EPS8, but different from EPS8L2, which is still present in stereocilia of *Myo15a^sh2 ^*mice (Furness *et al*. 2013).

## Discussion

The morphogenesis and maintenance of the stereociliary bundle in cochlear hair cells is a tightly regulated process requiring the interaction of several protein complexes, many of which include actin‐capping and ‐bundling proteins, as well as specialized myosin motors (Barr‐Gillespie, 2015). We found that the I‐BAR protein BAIAP2L2 (Ahmed *et al*. 2010) is a new hair bundle protein located at the tips of the two shorter rows of transducing stereocilia of cochlear IHCs and OHCs. In the absence of BAIAP2L2, the hair bundles of OHCs start to lose their third row of stereocilia just before the onset of hearing, which causes a significant reduction of the MET current. IHC stereociliary bundles appear to be unaffected at least until P49. By 8 months of age, both OHCs and IHCs have lost the third row of stereocilia and the second row was largely affected. The progressive deterioration of the stereociliary bundles in the absence of BAIAP2L2 leads to the rapid increase of hearing thresholds of *Baiap2l2^tm1b/tm1b^* mice, which become completely deaf before they reach 8 months of age (∼245 days). We also found that BAIAP2L2 interacts with other stereociliary proteins involved in the normal morphogenesis of the hair bundles (CDC42, RAC1, EPS8 and ESPNL) and that BAIAP2L2 localization to stereocilia tips depends on the motor protein MYO15A and its cargo EPS8 (Manor *et al*. 2011; Tadenev *et al*. 2019). We propose that BAIAP2L2 contributes to maintenance of the transducing stereocilia in mature cochlear hair cells by interacting with MYO15A and EPS8.

### *Baiap2l2* deficiency is associated with progressive hearing loss and the loss of the transducing stereocilia

*Baiap2l2*‐deficient mice show progressive hearing loss, which leads to a highly reduced hearing sensitivity over the entire frequency range investigated. At P14, ∼2 days after the onset of hearing in mice (Mikaelian & Ruben, 1964, Romand, 1983; Ehret, 1983), ABR thresholds were already significantly elevated in animals lacking BAIAP2L2, indicating that defects in hair cells probably begin during cochlear development. The initial morphogenesis of the stereocilia in *Baiap2l2^tm1b/tm1b^* mice appears normal because OHCs and IHCs acquired hair bundles with a normal staircase‐like structure. However, by the end of the second postnatal week, OHCs of *Baiap2l2^tm1b/tm1b^* mice start to lose the shortest (third) row of stereocilia. At this age, MET current and FM1‐43 uptake into the hair cells through the MET channels were also significantly reduced in the OHCs of these mice. By 8 months of age, both OHCs and IHCs from *Baiap2l2^tm1b/tm1b^* mice have lost most of the shorter transducing stereocilia (second and third rows). Altogether, these results indicate that BAIAP2L2 is essential for the maintenance of transducing stereocilia following morphogenesis.

Several proteins are known to regulate the length of the hair bundles during early stages of stereocilia growth through actin capping activity (Barr‐Gillespie, 2015). Some of these proteins appear to be particularly enriched in the shorter transducing stereocilia, including TWF2 (Peng *et al*. 2009), CAPZB (Avenarius *et al*. 2017) and EPS8L2 (Furness *et al*. 2013). EPS8L2 is a member of a family of actin‐regulatory proteins (EPS8 and EPS8L1‐L3) endowed with actin bundling and capping activities (Offenhäuser *et al*. 2004). In the hair cells, EPS8L2 acts by restricting the length of the shorter stereociliary rows by means of capping the barbed ends of actin filaments (Furness *et al*. 2013). In *Eps8l2* knockout mice, both IHCs and OHCs start to lose the third stereocilia row from ∼1 month of age and, by 8 months, they almost completely disappear (Furness *et al*. 2013). The loss of third row stereocilia has also been observed for mice lacking ESPNL, an actin‐regulating cargo of MYO3A/B (Ebrahim *et al*. 2016). In *Espnl* knockout mice, OHCs from the basal regions of cochleae begin to lose their third row of stereocilia starting from ∼P10, whereas OHCs from the more apical regions of the cochlea retained normal morphology (Ebrahim *et al*. 2016). Despite the common loss of third row stereocilia for *Baiap2l2*, *Eps8l2* and *Epsnl* knockout OHCs, the effect on hearing may not be uniform. *Espnl* and *Eps8l2* deficiency produce primarily high frequency hearing loss, whereas *Baiap2l2* deficiency appears to result in hearing loss over the entire mouse auditory frequency range. Despite the presence of BAIAP2L2 in IHCs from early stages of postnatal development, their hair bundle appeared unaffected at least up to P49. The lack of a significant IHC phenotype in *Baiap2l2* deficient mice until later in life could be a result of the presence of functional redundancy among I‐BAR proteins (see below).

### Functional maturation of the stereociliary bundle of cochlear hair cells

Our data indicate that hearing loss in *Baiap2l2* deficient mice is caused by the gradual loss of the shortest rows of transducing stereocilia, which apparently leads to a reduction in the number of functional MET channels. Although OHCs exhibited hair bundle defects already at pre‐hearing stages of development, by 8 months of age, both hair cell types showed substantial loss of row 2 and row 3 stereocilia. Several protein complexes are needed to achieve the extremely precise regulation of the stereocilia actin cytoskeleton required to establish the fine structure of the hair bundle. This includes the regulation of the stereocilia length, which is not only identical within rows and between neighbouring hair cells, but also changes along the cochlea and between rows within each bundle (Tilney *et al*. 1992; Kaltenbach *et al*. 1994; Reviewed by: Barr‐Gillespie, 2015; Vélez‐Ortega & Frolenkov, 2019). The widening of the stereocilia in both IHCs and OHCs depends on the ability of the actin‐bundling protein such as ESPN to bind to and cross‐link the actin filaments (Bartles *et al*. 1998; Sekerková *et al*. 2011). In adult cochlear hair cells, the tallest row of stereocilia contains a protein complex formed by EPS8, MYO15A‐S, WHRN, GPSM2 and GNAI3, which acts to identify, selectively lengthen, as well as widen row 1 relative to the other shorter rows (Belyantseva *et al*. 2003; Mburu *et al*. 2003; Manor *et al*. 2011; Zampini *et al*. 2011; Tarchini *et al*. 2016; Tadenev *et al*. 2019). Although the shaping of the shortest two rows of stereocilia is less well characterized, recent data indicate that EPS8L2 (Furness *et al*. 2013), MYO15A‐L (Fang *et al*. 2015: Krey *et al*. 2020), CAPZB (Avenarius *et al*. 2017), TWF2 (Peng *et al*. 2009) and gelsolin (Mburu *et al*. 2010; Olt *et al*. 2014) are all recruited to the tips of these stereocilia, where they may form a complex or complexes. The actin‐capping activity of EPS8L2, CAPZB and TWF2 inhibits actin polymerization, thus shifting the equilibrium in favour of depolymerization and resulting in their rows being shorter than row 1. The current data do not demonstrate that BAIAP2L2 interacts *in vivo* with any of the proteins thus far identified as regulators of row 2 and 3 stereocilia. However, as mentioned above, the progressive loss of row 3 stereocilia is shared by *Baiap2l2*, *Eps8l2* and *Espnl‐*deficient OHCs, suggesting that the complementary or co‐operative activity of these proteins may be required for maintenance of mature stereocilia. Whether these proteins interact in stereocilia remains to be determined. However, the finding that BAIAP2L2 binds EPSNL *in vitro*, together with the observation that localization that both BAIAP2L2 and ESPNL concentrate at the tips of row 2 stereocilia (Ebrahim *et al*. 2016), suggests that a stereociliary BAIAP2L2‐EPSNL interaction might be possible, although EPSNL and BAIAP2L2 do localize to stereocilia tips independently.

I‐BAR proteins are known to exhibit functional redundancy (Saarikangas *et al*. 2015; Chou *et al*. 2017). Therefore, the presence of compensatory I‐BAR protein members could explain why stereocilia morphogenesis in hair cells is normal despite BAIAP2L2 being expressed from early stages of postnatal development, and also why stereocilia are initially normal in IHCs that also localize BAIAP2L2 at their stereocilia tip. Notably, the I‐BAR proteins BAIAP2 and BAIAP2L1 are both detected in hair cells (umgear.org).

### BAIAP2L2 and protein trafficking within stereocilia

The exact mechanism of how the complex of MYO15A‐S and EPS8 facilitates the localization of BAIAP2L2 to the shorter two rows of stereocilia, and not the tallest row where the majority of the EPS8 signal resides, remains unknown. We hypothesize that BAIAP2L2 is trafficked to all stereocilia tips as a cargo of MYO15A‐EPS8‐BAIAP2L2, although it is selectively retained at the rows of the shorter tips by an unknown mechanism. The transduction channels are located selectively at stereocilia rows 2 and 3 (Beurg *et al*. 2009), and so retention of BAIAP2L2 may require the transduction channels themselves or a signal dependent on transduction. Why the MYO15A‐EPS8 complex accumulates at the tips of row 1 without the presence of BAIAP2L2 is unknown.

### I‐BAR proteins regulate actin protrusions

The I‐BAR proteins, by first dimerizing and then binding to the membrane, are capable of detecting and inducing negative membrane curvature, such as that present at the tip of a membrane protrusion (Zhao *et al*. 2011). Thus, by localizing to growing actin protrusions and recruiting proteins which facilitate actin filament elongation, members of this family are able to modulate protrusion growth (Sudhaharan *et al*. 2016). For example, BAIAP2L1 is responsible for localizing EPS8 to the growing microvilli of the brush border in the gut, and regulating their growth directly via its WH2 domain (Postema *et al*. 2018). BAIAP2L1 localizes itself via the I‐BAR domain, probably by detecting negative membrane curvature, and loss of the SH3 domain results in shortened microvilli and a complete lack of EPS8 localization (Postema *et al*. 2018).

BAIAP2L2 represents an exception among the I‐BAR proteins because it appears to show particularly high expression in epithelial cells (Carman & Dominguez, 2018) and has a relatively flat membrane‐binding surface; thus, instead of promoting membrane protrusion or invagination, it assembles into planar membrane sheets (Pykäläinen *et al*. 2011). The diameters of cochlear stereocilia are relatively large compared to microvilli or filopodia; however, an I‐BAR protein favouring a flat membrane may readily associate at stereocilia tips. In the OHC stereocilia of the cochlea, the MYO15A‐S‐EPS8 complex precedes BAIAP2L2 at the tips of the shorter rows of stereocilia, unlike that seen with BAIAP2L1 and MYO15A at the brush border of the gut. Since expression of *Baiap2l2 *lags behind that of *Myo15a* and *Eps8* (Scheffer *et al*. 2015), when *Baiap2l2* finally appears, it is then transported to stereocilia tips via the MYO15A‐S‐EPS8 complex by an interaction that likely requires SH3 domain‐dependent inretaction of BAIAP2L2 and EPS8. Because the protein does not function to detect negative membrane curvature in this setting (Pykäläinen *et al*. 2011), it probably modulates actin dynamics directly after recruitment via the WH2 domain or indirectly through the recruitment or stabilization of additional currently unknown actin effector proteins (Zhao *et al*. 2011, Postema *et al*. 2018).

## Additional information

### Competing interests

The authors declare that they have no competing interests.

### Author contributions

All authors helped with the collection, analysis and interpretation of the data, and commented on the manuscript. AJC, JH, PGB‐G and WM wrote the paper. SDMB, MRB and WM conceived the study in the UK; PGB‐G conceived the study in the USA. PGB‐G and WM co‐ordinated the study. All authors approved the final version of the article submitted for publication. All authors agree to be accountable for all aspects of the work in ensuring that questions related to the accuracy or integrity of any part of the work are appropriately investigated and resolved. All persons designated as authors qualify for authorship, and all those who qualify for authorship are listed.

### Funding

This work was supported by the: BBSRC (BB/S006257/1) to SDMB, MRB and WM and National Institutes of Health grant R01DC002368 to PGB‐G. AJC is funded by a PhD studentship from Action on Hearing Loss (S50).

## Supporting information


**Statistical Summary Document**

**Data S1**

## Data Availability

The data that support the findings of this study are available from the corresponding authors upon reasonable request.
