# Supplementary material for: Loss of Baiap2l2 destabilizes the transducing stereocilia of cochlear hair cells and leads to deafness
Source: J Physiol. 2020 Nov 26;599(4):1173–98. doi: 10.1113/JP280670 (PMC7898316; doi:10.1113/JP280670)
Supplement: Supplementary file 1 — Statistical Summary Document [file TJP-599-1173-s001.pdf]

The Journal of Physiology

Statistical Summary Document

Manuscript Title: Loss of *Baiap2l2* destabilizes the transducing stereocilia of cochlear hair cells and leads to deafness

Authors: Adam J. Carlton, Julia Halford, Anna Underhill, Jing-Yi Jeng, Matthew R. Avenarius, Merle L. Gilbert, Federico Ceriani, Kimimuepigha Ebisine, Steve D. M. Brown, Michael R. Bowl, Peter G. Barr-Gillespie, Walter Marcotti

Animal model used, if applicable:
Mouse– *Baiap2l2*$^{tm1b/+}$, *Baiap2l2*$^{tm1b/tm1b}$, *Baiap2l2*$^{\Delta16/\Delta16}$, *Baiap2l2*$^{+/\Delta16}$ and *Baiap2l2*$^{+/+}$

Underlying hypothesis:
This study investigates the hypothesis that the loss of BAIAP2L2 causes defects in stereocilia and leads to deafness.

Definitions of 'n':
Question 1-5: n = number of animals
Question 6-9: n = number of stereocilia
Question 10-17: n = number of cells

Mice were from both sexes unless otherwise stated

Statistical summary table:

Experimental question number*	Finding/ conclusion	Experimental location/ variable e.g. cortex vs cerebellum or genotype	Mean or Median value	SD or MAD	n (value)	P	Units	Data comparisons e.g. WT vs KO	Statistical test	Any other variable e.g. subjects' age or sex	Figure/table in which data are presented	Comments e.g. observation
1. Click-evoked ABR thresholds	No ABR threshold difference in *Baiap2l2*$^{tm1b/+}$ from between P19 and P245.	*Baiap2l2*$^{tm1b/+}$ P14	61.67	2.58	6	< 0.0001	dB	All ages	1-way ANOVA	14-245 days	2A	
		Baiap2l2$^{tm1b/+}$ P19-22	42.50	1.14	14							
		Baiap2l2$^{tm1b/+}$ P48-53	40.63	3.20	8							
		Baiap2l2$^{tm1b/+}$ P104-116	39.00	2.24	6							
		Baiap2l2$^{tm1b/+}$ P166-245	39.17	2.04	6							
		Statistical tests for the above data from *Baiap2l2*$^{tm1b/+}$.				0.1357		All ages above P19	1-way ANOVA			

						< 0.0001		P14 vs. P19-22	1-way ANOVA, Tukey's post-test			
		Baiap2l2$^{tm1b/tm1b}$ P14	85.56	6.35	9	< 0.0001	dB	All ages	1-way ANOVA	P14-P245		
		Baiap2l2$^{tm1b/tm1b}$ P19-22	69.64	1.23	14							
		Baiap2l2$^{tm1b/tm1b}$ P48-53	80.00	6.45	7							
		Baiap2l2$^{tm1b/tm1b}$ P104-116	95.83	4.92	6							
		Baiap2l2$^{tm1b/tm1b}$ P166-245	100.00	0.00	6							
		Statistical tests for the above data from Baiap2l2tm1b/tm1b				< 0.0001		P14 vs. P19-22	1-way ANOVA, Tukey's post-test			
						0.2185		P14 vs. P48-61				
		Statistical tests for the above data from Baiap2l2tm1b/tm1b and Baiap2l2tm1b/tm1b				< 0.0001		All ages	2-way ANOVA			
2. Pure-tone ABR thresholds	Elevated in *Baiap2l2*$^{tm1b/tm1b}$.	*Baiap2l2*$^{tm1b/+}$ 3 kHz	86.67	2.58	6		dB	P14		P14-P245	2B, 2C	
		Baiap2l2$^{tm1b/+}$ 6 kHz	75.00	3.16	6							
		Baiap2l2$^{tm1b/+}$ 12 kHz	45.00	6.32	6							
		Baiap2l2$^{tm1b/+}$ 18 kHz	37.50	6.89	6							
		Baiap2l2$^{tm1b/+}$ 24 kHz	35.00	6.12	5							
		Baiap2l2$^{tm1b/+}$ 30 kHz	48.00	6.71	5							

		Baiap2l2$^{tm1b/+}$ 36 kHz	67.00	7.58	5							
		Baiap2l2$^{tm1b/+}$ 3 kHz	80.71	1.37	14							
		Baiap2l2$^{tm1b/+}$ 6 kHz	46.79	0.66	14							
		Baiap2l2$^{tm1b/+}$ 12 kHz	23.93	1.30	14							
		Baiap2l2$^{tm1b/+}$ 18 kHz	24.29	1.16	14			P19-22				
		Baiap2l2$^{tm1b/+}$ 24 kHz	30.00	1.05	14							
		Baiap2l2$^{tm1b/+}$ 30 kHz	36.43	0.82	14							
		Baiap2l2$^{tm1b/+}$ 36 kHz	44.29	1.03	14							
		Baiap2l2$^{tm1b/+}$ 3 kHz	70.00	6.55	8							
		Baiap2l2$^{tm1b/+}$ 6 kHz	38.13	2.59	8							
		Baiap2l2$^{tm1b/+}$ 12 kHz	21.25	3.54	8							
		Baiap2l2$^{tm1b/+}$ 18 kHz	24.38	4.17	8			P48-53				
		Baiap2l2$^{tm1b/+}$ 24 kHz	35.00	4.63	8							
		Baiap2l2$^{tm1b/+}$ 30 kHz	50.71	6.73	7							
		Baiap2l2$^{tm1b/+}$ 36 kHz	86.43	9.00	7							
		Baiap2l2$^{tm1b/+}$ 3 kHz	39.00	2.24	5			P104-116				
		Baiap2l2$^{tm1b/+}$	62.00	5.70	5							

		6 kHz									
		Baiap2l2$^{tm1b/+}$ 12 kHz	43.00	4.47	5						
		Baiap2l2$^{tm1b/+}$ 18 kHz	20.00	0.00	5						
		Baiap2l2$^{tm1b/+}$ 24 kHz	25.00	3.54	5						
		Baiap2l2$^{tm1b/+}$ 30 kHz	39.00	5.48	5						
		Baiap2l2$^{tm1b/+}$ 36 kHz	93.00	8.37	5						
		Baiap2l2$^{tm1b/+}$ 3 kHz	76.67	4.08	6			P166-245			
		Baiap2l2$^{tm1b/+}$ 6 kHz	38.33	4.08	6						
		Baiap2l2$^{tm1b/+}$ 12 kHz	20.83	3.76	6						
		Baiap2l2$^{tm1b/+}$ 18 kHz	30.83	8.01	6						
		Baiap2l2$^{tm1b/+}$ 24 kHz	88.33	6.83	6						
		Baiap2l2$^{tm1b/+}$ 30 kHz	98.33	2.58	6						
		Baiap2l2$^{tm1b/+}$ 36 kHz	95.00	0.00	6						
		Baiap2l2$^{tm1b/tm1b}$ 3 kHz	93.33	2.50	9			P14			
		Baiap2l2$^{tm1b/tm1b}$ 6 kHz	76.11	4.86	9						
		Baiap2l2$^{tm1b/tm1b}$ 12 kHz	58.33	6.12	9						
		Baiap2l2$^{tm1b/tm1b}$ 18 kHz	58.89	10.24	9						

		Baiap2l2$^{tm1b/tm1b}$ 24 kHz	66.11	11.40	9							
		Baiap2l2$^{tm1b/tm1b}$ 30 kHz	83.89	11.67	9							
		Baiap2l2$^{tm1b/tm1b}$ 36 kHz	97.22	2.64	9							
		Baiap2l2$^{tm1b/tm1b}$ 3 kHz	85.00	1.48	14			P19-22				
		Baiap2l2$^{tm1b/tm1b}$ 6 kHz	60.00	1.39	14							
		Baiap2l2$^{tm1b/tm1b}$ 12 kHz	52.50	2.95	14							
		Baiap2l2$^{tm1b/tm1b}$ 18 kHz	68.21	2.20	14							
		Baiap2l2$^{tm1b/tm1b}$ 24 kHz	78.93	1.30	14							
		Baiap2l2$^{tm1b/tm1b}$ 30 kHz	86.79	1.00	14							
		Baiap2l2$^{tm1b/tm1b}$ 36 kHz	93.57	0.82	14							
		Baiap2l2$^{tm1b/tm1b}$ 3 kHz	80.71	3.45	7			P48-53				
		Baiap2l2$^{tm1b/tm1b}$ 6 kHz	65.00	8.16	7							
		Baiap2l2$^{tm1b/tm1b}$ 12 kHz	62.86	11.85	7							
		Baiap2l2$^{tm1b/tm1b}$ 18 kHz	80.71	10.97	7							
		Baiap2l2$^{tm1b/tm1b}$ 24 kHz	96.43	5.56	7							
		Baiap2l2$^{tm1b/tm1b}$ 30 kHz	100.00	0.00	7							
		Baiap2l2$^{tm1b/tm1b}$	100.00	0.00	7							

		36 kHz										
		Baiap2l2$^{tm1b/tm1b}$ 3 kHz	88.33	5.16	6			P104-116				
		Baiap2l2$^{tm1b/tm1b}$ 6 kHz	80.00	6.32	6							
		Baiap2l2$^{tm1b/tm1b}$ 12 kHz	88.33	10.3	6							
		Baiap2l2$^{tm1b/tm1b}$ 18 kHz	100.00	0.00	6							
		Baiap2l2$^{tm1b/tm1b}$ 24 kHz	100.00	0.00	6							
		Baiap2l2$^{tm1b/tm1b}$ 30 kHz	100.00	0.00	6							
		Baiap2l2$^{tm1b/tm1b}$ 36 kHz	100.00	0.00	6							
		Baiap2l2$^{tm1b/tm1b}$ 3 kHz	100.00	0.00	6			P166-245				
		Baiap2l2$^{tm1b/tm1b}$ 6 kHz	100.00	0.00	6							
		Baiap2l2$^{tm1b/tm1b}$ 12 kHz	100.00	0.00	6							
		Baiap2l2$^{tm1b/tm1b}$ 18 kHz	100.00	0.00	6							
		Baiap2l2$^{tm1b/tm1b}$ 24 kHz	100.00	0.00	6							
		Baiap2l2$^{tm1b/tm1b}$ 30 kHz	100.00	0.00	6							
		Baiap2l2$^{tm1b/tm1b}$ 36 kHz	100.00	0.00	6							
		Statistical tests for the above data				< 0.0001		*Baiap2l2*$^{tm1b/+}$ vs. *Baiap2l2*$^{tm1b/tm1b}$ at P14	2-way ANOVA			

			< 0.0001		*Baiap2l2*$^{tm1b/+}$ vs. *Baiap2l2*$^{tm1b/tm1b}$ at P19-22	2-way ANOVA			
			< 0.0001		*Baiap2l2*$^{tm1b/+}$ vs. *Baiap2l2*$^{tm1b/tm1b}$ at P48-53	2-way ANOVA			
			< 0.0001		*Baiap2l2*$^{tm1b/+}$ vs. *Baiap2l2*$^{tm1b/tm1b}$ at P104-116	2-way ANOVA			
			< 0.0001		*Baiap2l2*$^{tm1b/+}$ vs. *Baiap2l2*$^{tm1b/tm1b}$ at P166-245	2-way ANOVA			
3. Wave 1 amplitude and latency	Wave 1 amplitude and latency were different between *Baiap2l2*$^{tm1b/tm1b}$ and *Baiap2l2*$^{tm1b/tm1b}$	Wave 1 amplitude.	< 0.0001		*Baiap2l2*$^{tm1b/+}$ vs. *Baiap2l2*$^{tm1b/tm1b}$	2-way ANOVA	P19-53	2F	
			< 0.0001		*Baiap2l2*$^{tm1b/+}$ vs. *Baiap2l2*$^{tm1b/tm1b}$	2-way ANOVA, Tukey's post-test	P19-22		
			< 0.0001		*Baiap2l2*$^{tm1b/+}$ vs. *Baiap2l2*$^{tm1b/tm1b}$		P48-53		
		Wave 1 latency.	0.0247		*Baiap2l2*$^{tm1b/+}$ vs. *Baiap2l2*$^{tm1b/tm1b}$	2-way ANOVA	P19-53		
			< 0.0001		*Baiap2l2*$^{tm1b/+}$ vs. *Baiap2l2*$^{tm1b/tm1b}$	2-way ANOVA, Tukey's post-test	P19-22		
			< 0.0001		*Baiap2l2*$^{tm1b/+}$ vs. *Baiap2l2*$^{tm1b/tm1b}$		P48-53		
4. Wave 1 amplitude and latency relative to threshol	Wave 1 amplitude was different between *Baiap2l2*^{tm1}	Wave 1 amplitude relative to threshold.	< 0.0001		*Baiap2l2*$^{tm1b/+}$ vs. *Baiap2l2*$^{tm1b/tm1b}$	2-way ANOVA	P19-53	2G	
			< 0.0001		*Baiap2l2*$^{tm1b/+}$ vs. *Baiap2l2*$^{tm1b/tm1b}$	2-way ANOVA, Tukey's post-test	P19-22		
			0.0468		*Baiap2l2*$^{tm1b/+}$ vs. *Baiap2l2*$^{tm1b/tm1b}$		P48-53		

	$^{b/tm1b}$ and *Baiap2l2*$^{tm1b/tm1b}$	Wave 1 latency relative to threshold.				0.9677		*Baiap2l2*$^{tm1b/+}$ vs. *Baiap2l2*$^{tm1b/tm1b}$	2-way ANOVA	P19-53		
5. DPOAE threshold	DPOAE thresholds in *Baiap2l2*$^{tm1b/tm1b}$ mice were raised compared to *Baiap2l2*$^{tm1b/+}$	*Baiap2l2*$^{tm1b/+}$ 6 kHz	75	5	16	< 0.0001	dB	*Baiap2l2*$^{tm1b/+}$ vs. *Baiap2l2*$^{tm1b/tm1b}$	aligned ranks transformation 2-way ANOVA	P19-61	3A,D	Data are median and MAD
		Baiap2l2$^{tm1b/+}$ 12 kHz	40	0	16							
		Baiap2l2$^{tm1b/+}$ 18 kHz	50	10	16							
		Baiap2l2$^{tm1b/+}$ 24 kHz	50	10	16							
		Baiap2l2$^{tm1b/tm1b}$ 6 kHz	85	5	18							
		Baiap2l2$^{tm1b/tm1b}$ 12 kHz	70	10	18							
		Baiap2l2$^{tm1b/tm1b}$ 18 kHz	80	10	18							
		Baiap2l2$^{tm1b/tm1b}$ 24 kHz	75	15	18							
		Baiap2l2$^{tm1b/+}$ 6 kHz	80	10	5	< 0.0001				P104-116	3B,E	
		Baiap2l2$^{tm1b/+}$ 12 kHz	30	0	5							
		Baiap2l2$^{tm1b/+}$ 18 kHz	50	0	5							
		Baiap2l2$^{tm1b/+}$ 24 kHz	60	10	5							
		Baiap2l2$^{tm1b/tm1b}$ 6 kHz	90	0	6							
		Baiap2l2$^{tm1b/tm1b}$ 12 kHz	70	10	6							
		Baiap2l2$^{tm1b/tm1b}$ 18 kHz	90	0	6							
		Baiap2l2$^{tm1b/tm1b}$ 24 kHz	90	0	6							
		Baiap2l2$^{tm1b/+}$ 6 kHz	70	0	6	< 0.0001				P166-245	3C,F	
		Baiap2l2$^{tm1b/+}$ 12 kHz	40	5	6							
		Baiap2l2$^{tm1b/+}$ 18 kHz	70	20	6							

		Baiap2l2$^{tm1b/+}$ 24 kHz	90	0	6							
		Baiap2l2$^{tm1b/tm1b}$ 6 kHz	80	10	6							
		Baiap2l2$^{tm1b/tm1b}$ 12 kHz	90	0	6							
		Baiap2l2$^{tm1b/tm1b}$ 18 kHz	90	0	6							
		Baiap2l2$^{tm1b/tm1b}$ 24 kHz	90	0	6							
6. IHC stereocilia length	No consistent trend in the direction of the differences.	Row 1 *Baiap2l2*$^{\Delta16/+}$	3.20	0.35	42	0.6481	µm	*Baiap2l2*$^{\Delta16/+}$ vs. *Baiap2l2*$^{\Delta16/\Delta16}$	t-test	P0.5	7A	
		Row 1 *Baiap2l2*$^{\Delta16/\Delta16}$	3.25	0.35	12							
		Row 1 *Baiap2l2*$^{\Delta16/+}$	3.14	0.43	15	0.1571		*Baiap2l2*$^{\Delta16/+}$ vs. *Baiap2l2*$^{\Delta16/\Delta16}$		P3.5	7B	
		Row 1 *Baiap2l2*$^{\Delta16/\Delta16}$	3.33	0.46	42							
		Row 1 *Baiap2l2*$^{+/+}$	2.84	0.24	9	0.0705		*Baiap2l2*$^{+/+}$ vs. *Baiap2l2*$^{\Delta16/\Delta16}$		P5.5	7C	
		Row 1 *Baiap2l2*$^{\Delta16/\Delta16}$	3.07	0.32	16							
		Row 1 *Baiap2l2*$^{+/+}$	3.35	0.35	13	0.0506		*Baiap2l2*$^{+/+}$ vs. *Baiap2l2*$^{\Delta16/\Delta16}$		P8.5	7D	
		Row 1 *Baiap2l2*$^{\Delta16/\Delta16}$	3.64	0.42	15							
		Row 1 *Baiap2l2*$^{\Delta16/+}$	6.14	0.67	4	0.7017		*Baiap2l2*$^{\Delta16/+}$ vs. *Baiap2l2*$^{\Delta16/\Delta16}$		P14.5	7E	
		Row 1 *Baiap2l2*$^{\Delta16/\Delta16}$	6.26	0.48	12							
		Row 1 *Baiap2l2*$^{+/+}$	6.50	0.24	9	0.1664		*Baiap2l2*$^{+/+}$ vs. *Baiap2l2*$^{\Delta16/\Delta16}$		P21	7F	
		Row 1 *Baiap2l2*$^{\Delta16/\Delta16}$	6.13	0.75	10							
		Row 2 *Baiap2l2*$^{\Delta16/+}$	2.55	0.34	42	0.7933		*Baiap2l2*$^{\Delta16/+}$ vs. *Baiap2l2*$^{\Delta16/\Delta16}$		P0.5	7A	
		Row 2 *Baiap2l2*$^{\Delta16/\Delta16}$	2.58	0.29	12							
		Row 2 *Baiap2l2*$^{\Delta16/+}$	2.16	0.35	15	0.2737		*Baiap2l2*$^{\Delta16/+}$ vs. *Baiap2l2*$^{\Delta16/\Delta16}$		P3.5	7B	
		Row 2 *Baiap2l2*$^{\Delta16/\Delta16}$	2.05	0.34	42							
		Row 2 *Baiap2l2*$^{+/+}$	2.01	0.22	9	0.3790		*Baiap2l2*$^{+/+}$ vs. *Baiap2l2*$^{\Delta16/\Delta16}$		P5.5	7C	
		Row 2 *Baiap2l2*$^{\Delta16/\Delta16}$	1.90	0.34	16							
		Row 2 *Baiap2l2*$^{+/+}$	2.24	0.26	13	0.0003		*Baiap2l2*$^{+/+}$ vs. *Baiap2l2*$^{\Delta16/\Delta16}$		P8.5	7D	
		Row 2 *Baiap2l2*$^{\Delta16/\Delta16}$	1.84	0.25	15							
		Row 2 *Baiap2l2*$^{\Delta16/+}$	2.18	0.33	4	0.1657				P14.5	7E	

		Row 2 *Baiap2l2*$^{\Delta16/\Delta16}$	2.41	0.26	12			*Baiap2l2*$^{\Delta16/+}$ vs. *Baiap2l2*$^{\Delta16/\Delta16}$				
		Row 2 *Baiap2l2*$^{+/+}$	2.27	0.27	9	0.7738		*Baiap2l2*$^{+/+}$ vs. *Baiap2l2*$^{\Delta16/\Delta16}$		P21	7F	
		Row 2 *Baiap2l2*$^{\Delta16/\Delta16}$	2.30	0.29	10							
7. IHC stereocilia width	No consistent trend in the direction of the differences.	Row 1 *Baiap2l2*$^{\Delta16/+}$	0.33	0.04	42	0.0251	µm	*Baiap2l2*$^{\Delta16/+}$ vs. *Baiap2l2*$^{\Delta16/\Delta16}$	t-test	P0.5	7G	
		Row 1 *Baiap2l2*$^{\Delta16/\Delta16}$	0.30	0.03	12							
		Row 1 *Baiap2l2*$^{\Delta16/+}$	0.46	0.06	15	< 0.001		*Baiap2l2*$^{\Delta16/+}$ vs. *Baiap2l2*$^{\Delta16/\Delta16}$		P3.5	7H	
		Row 1 *Baiap2l2*$^{\Delta16/\Delta16}$	0.56	0.06	42							
		Row 1 *Baiap2l2*$^{+/+}$	0.43	0.02	9	< 0.001		*Baiap2l2*$^{+/+}$ vs. *Baiap2l2*$^{\Delta16/\Delta16}$		P5.5	7I	
		Row 1 *Baiap2l2*$^{\Delta16/\Delta16}$	0.58	0.07	16							
		Row 1 *Baiap2l2*$^{+/+}$	0.64	0.06	13	0.1010		*Baiap2l2*$^{+/+}$ vs. *Baiap2l2*$^{\Delta16/\Delta16}$		P8.5	7J	
		Row 1 *Baiap2l2*$^{\Delta16/\Delta16}$	0.68	0.07	15							
		Row 1 *Baiap2l2*$^{\Delta16/+}$	0.79	0.04	4	0.0326		*Baiap2l2*$^{\Delta16/+}$ vs. *Baiap2l2*$^{\Delta16/\Delta16}$		P14.5	7K	
		Row 1 *Baiap2l2*$^{\Delta16/\Delta16}$	0.87	0.07	12							
		Row 1 *Baiap2l2*$^{+/+}$	0.64	0.03	9	0.2048		*Baiap2l2*$^{+/+}$ vs. *Baiap2l2*$^{\Delta16/\Delta16}$		P21	7L	
		Row 1 *Baiap2l2*$^{\Delta16/\Delta16}$	0.67	0.04	10							
		Row 2 *Baiap2l2*$^{\Delta16/+}$	0.32	0.04	42	0.0009		*Baiap2l2*$^{\Delta16/+}$ vs. *Baiap2l2*$^{\Delta16/\Delta16}$		P0.5	7G	
		Row 2 *Baiap2l2*$^{\Delta16/\Delta16}$	0.28	0.03	12							
		Row 2 *Baiap2l2*$^{\Delta16/+}$	0.57	0.06	15	0.3067		*Baiap2l2*$^{\Delta16/+}$ vs. *Baiap2l2*$^{\Delta16/\Delta16}$		P3.5	7H	
		Row 2 *Baiap2l2*$^{\Delta16/\Delta16}$	0.60	0.08	42							
		Row 2 *Baiap2l2*$^{+/+}$	0.51	0.05	9	0.0136		*Baiap2l2*$^{+/+}$ vs. *Baiap2l2*$^{\Delta16/\Delta16}$		P5.5	7I	
		Row 2 *Baiap2l2*$^{\Delta16/\Delta16}$	0.60	0.09	16							
		Row 2 *Baiap2l2*$^{+/+}$	0.67	0.07	13	0.0158		*Baiap2l2*$^{+/+}$ vs. *Baiap2l2*$^{\Delta16/\Delta16}$		P8.5	7J	
		Row 2 *Baiap2l2*$^{\Delta16/\Delta16}$	0.57	0.12	15							
		Row 2 *Baiap2l2*$^{\Delta16/+}$	0.59	0.11	4	0.0551		*Baiap2l2*$^{\Delta16/+}$ vs. *Baiap2l2*$^{\Delta16/\Delta16}$		P14.5	7K	
		Row 2 *Baiap2l2*$^{\Delta16/\Delta16}$	0.68	0.07	12							
		Row 2 *Baiap2l2*$^{+/+}$	0.51	0.06	9	0.7722		*Baiap2l2*$^{+/+}$ vs. *Baiap2l2*$^{\Delta16/\Delta16}$		P21	7L	
		Row 2 *Baiap2l2*$^{\Delta16/\Delta16}$	0.52	0.06	10							
		Row 1 *Baiap2l2*$^{+/+}$	2.73	0.30	13	0.0804	µm	*Baiap2l2*$^{+/+}$ vs. *Baiap2l2*$^{\Delta16/\Delta16}$	t-test	P5.5	7M	
		Row 1 *Baiap2l2*$^{\Delta16/\Delta16}$	2.88	0.19	23							

8. OHC stereocilia length		Row 1 *Baiap2l2*$^{\Delta16/+}$	3.00	0.17	73	0.0289		*Baiap2l2*$^{\Delta16/+}$ vs. *Baiap2l2*$^{\Delta16/\Delta16}$	t-test	P8.5	7N	
		Row 1 *Baiap2l2*$^{\Delta16/\Delta16}$	3.11	0.38	45							
		Row 1 *Baiap2l2*$^{+/+}$	2.84	0.20	31	0.0598		*Baiap2l2*$^{+/+}$ vs. *Baiap2l2*$^{\Delta16/\Delta16}$	t-test	P14.5	7O	
		Row 1 *Baiap2l2*$^{\Delta16/\Delta16}$	2.96	0.29	38							
		Row 1 *Baiap2l2*$^{+/+}$	3.16	1.14	12	0.8971		*Baiap2l2*$^{+/+}$ vs. *Baiap2l2*$^{\Delta16/\Delta16}$	t-test	P21	7P	
		Row 1 *Baiap2l2*$^{\Delta16/\Delta16}$	3.20	0.13	15							
		Row 2 *Baiap2l2*$^{+/+}$	2.12	0.27	13	0.0384		*Baiap2l2*$^{+/+}$ vs. *Baiap2l2*$^{\Delta16/\Delta16}$		P5.5	7M	
		Row 2 *Baiap2l2*$^{\Delta16/\Delta16}$	2.32	0.27	23							
		Row 2 *Baiap2l2*$^{\Delta16/+}$	1.90	0.22	73	0.0090		*Baiap2l2*$^{\Delta16/+}$ vs. *Baiap2l2*$^{\Delta16/\Delta16}$		P8.5	7N	
		Row 2 *Baiap2l2*$^{\Delta16/\Delta16}$	1.79	0.23	45							
		Row 2 *Baiap2l2*$^{+/+}$	2.02	0.19	31	0.2298		*Baiap2l2*$^{+/+}$ vs. *Baiap2l2*$^{\Delta16/\Delta16}$		P14.5	7O	
		Row 2 *Baiap2l2*$^{\Delta16/\Delta16}$	1.96	0.21	38							
		Row 2 *Baiap2l2*$^{+/+}$	1.90	0.67	12	0.2183		*Baiap2l2*$^{+/+}$ vs. *Baiap2l2*$^{\Delta16/\Delta16}$		P21	7P	
		Row 2 *Baiap2l2*$^{\Delta16/\Delta16}$	2.12	0.16	15							
		Row 3 *Baiap2l2*$^{+/+}$	1.07	0.17	3	0.0814		*Baiap2l2*$^{+/+}$ vs. *Baiap2l2*$^{\Delta16/\Delta16}$		P5.5	7M	
		Row 3 *Baiap2l2*$^{\Delta16/\Delta16}$	1.32	0.21	10							
		Row 3 *Baiap2l2*$^{\Delta16/+}$	1.25	0.15	64	< 0.0001		*Baiap2l2*$^{\Delta16/+}$ vs. *Baiap2l2*$^{\Delta16/\Delta16}$		P8.5	7N	
		Row 3 *Baiap2l2*$^{\Delta16/\Delta16}$	1.07	0.13	17							
		Row 3 *Baiap2l2*$^{+/+}$	1.28	0.20	22	0.2994		*Baiap2l2*$^{+/+}$ vs. *Baiap2l2*$^{\Delta16/\Delta16}$		P14.5	7O	
		Row 3 *Baiap2l2*$^{\Delta16/\Delta16}$	1.18	0.17	6							
		Row 3 *Baiap2l2*$^{+/+}$	0.98	0.25	9	-		*Baiap2l2*$^{+/+}$ vs. *Baiap2l2*$^{\Delta16/\Delta16}$	-	P21	7P	
		Row 3 *Baiap2l2*$^{\Delta16/\Delta16}$	1.35	N/A	1							
9. OHC stereocilia width		Row 1 *Baiap2l2*$^{+/+}$	0.32	0.03	13	0.0023	µm	*Baiap2l2*$^{+/+}$ vs. *Baiap2l2*$^{\Delta16/\Delta16}$	t-test	P5.5	7Q	
		Row 1 *Baiap2l2*$^{\Delta16/\Delta16}$	0.28	0.03	23							
		Row 1 *Baiap2l2*$^{\Delta16/+}$	0.35	0.03	73	< 0.0001		*Baiap2l2*$^{\Delta16/+}$ vs. *Baiap2l2*$^{\Delta16/\Delta16}$		P8.5	7R	
		Row 1 *Baiap2l2*$^{\Delta16/\Delta16}$	0.38	0.04	45							
		Row 1 *Baiap2l2*$^{+/+}$	0.34	0.04	31	0.2820		*Baiap2l2*$^{+/+}$ vs. *Baiap2l2*$^{\Delta16/\Delta16}$		P14.5	7S	
		Row 1 *Baiap2l2*$^{\Delta16/\Delta16}$	0.35	0.04	38							
		Row 1 *Baiap2l2*$^{+/+}$	0.30	0.03	12	0.7075		*Baiap2l2*$^{+/+}$ vs. *Baiap2l2*$^{\Delta16/\Delta16}$		P21	7T	
		Row 1 *Baiap2l2*$^{\Delta16/\Delta16}$	0.30	0.04	15							

		Row 2 *Baiap2l2*$^{+/+}$	0.40	0.06	13	0.1253		*Baiap2l2*$^{+/+}$ vs. *Baiap2l2*$^{\Delta16/\Delta16}$		P5.5	7Q	
		Row 2 *Baiap2l2*$^{\Delta16/\Delta16}$	0.38	0.03	23							
		Row 2 *Baiap2l2*$^{\Delta16/+}$	0.37	0.03	73	0.0214		*Baiap2l2*$^{\Delta16/+}$ vs. *Baiap2l2*$^{\Delta16/\Delta16}$		P8.5	7R	
		Row 2 *Baiap2l2*$^{\Delta16/\Delta16}$	0.38	0.04	45							
		Row 2 *Baiap2l2*$^{+/+}$	0.41	0.04	31	0.8592		*Baiap2l2*$^{+/+}$ vs. *Baiap2l2*$^{\Delta16/\Delta16}$		P14.5	7S	
		Row 2 *Baiap2l2*$^{\Delta16/\Delta16}$	0.41	0.04	38							
		Row 2 *Baiap2l2*$^{+/+}$	0.36	0.02	12	0.0094		*Baiap2l2*$^{+/+}$ vs. *Baiap2l2*$^{\Delta16/\Delta16}$		P21	7T	
		Row 2 *Baiap2l2*$^{\Delta16/\Delta16}$	0.32	0.04	15							
		Row 3 *Baiap2l2*$^{+/+}$	0.27	0.10	3	0.1143		*Baiap2l2*$^{+/+}$ vs. *Baiap2l2*$^{\Delta16/\Delta16}$		P5.5	7Q	
		Row 3 *Baiap2l2*$^{\Delta16/\Delta16}$	0.21	0.04	10							
		Row 3 *Baiap2l2*$^{\Delta16/+}$	0.29	0.04	64	0.4959		*Baiap2l2*$^{\Delta16/+}$ vs. *Baiap2l2*$^{\Delta16/\Delta16}$		P8.5	7R	
		Row 3 *Baiap2l2*$^{\Delta16/\Delta16}$	0.28	0.05	17							
		Row 3 *Baiap2l2*$^{+/+}$	0.34	0.05	22	0.0470		*Baiap2l2*$^{+/+}$ vs. *Baiap2l2*$^{\Delta16/\Delta16}$		P14.5	7S	
		Row 3 *Baiap2l2*$^{\Delta16/\Delta16}$	0.30	0.03	6							
		Row 3 *Baiap2l2*$^{+/+}$	0.26	0.06	9	-		-	-	P21	7T	
		Row 3 *Baiap2l2*$^{\Delta16/\Delta16}$	0.20	N/A	1							
10. OHC maximal MET current at -124 mV	MET currents were smaller in *Baiap2l2*$^{tm1b/tm1b}$ at P11	*Baiap2l2*$^{tm1b/+}$	-915	145	11	0.1711	pA	*Baiap2l2*$^{tm1b/tm1b}$ vs. *Baiap2l2*$^{tm1b/+}$	t-test	P8-9	8G	
		Baiap2l2$^{tm1b/tm1b}$	-767	327	17							
		Baiap2l2$^{tm1b/+}$	-1200	317	6	0.0036		*Baiap2l2*$^{tm1b/tm1b}$ vs. *Baiap2l2*$^{tm1b/+}$		P11		
		Baiap2l2$^{tm1b/tm1b}$	-511	131	4							
11. OHC maximal MET current at +96 mV	MET currents were smaller in *Baiap2l2*$^{tm1b/tm1b}$ at P11	*Baiap2l2*$^{tm1b/+}$	955	206	11	0.2020	pA	*Baiap2l2*$^{tm1b/tm1b}$ vs. *Baiap2l2*$^{tm1b/+}$	t-test	P8-9	8H	
		Baiap2l2$^{tm1b/tm1b}$	805	337	16							
		Baiap2l2$^{tm1b/+}$	1484	205	5	< 0.0001		*Baiap2l2*$^{tm1b/tm1b}$ vs. *Baiap2l2*$^{tm1b/+}$		P11		
		Baiap2l2$^{tm1b/tm1b}$	579	79	4							
12. OHC MET	No difference	*Baiap2l2*$^{tm1b/+}$	0.065	0.028	11	0.1035		*Baiap2l2*$^{tm1b/tm1b}$ vs. *Baiap2l2*$^{tm1b/+}$	t-test	P8-9	8I	
		Baiap2l2$^{tm1b/tm1b}$	0.113	0.090	17							

channel open probability at -124 mV	between *Baiap2l2*$^{tm1b/tm1b}$ and control.	*Baiap2l2*$^{tm1b/+}$	0.085	0.017	6	0.1950		*Baiap2l2*$^{tm1b/tm1b}$ vs. *Baiap2l2*$^{tm1b/+}$		P11		
		Baiap2l2$^{tm1b/tm1b}$	0.122	0.063	4							
13. OHC MET channel open probability at +96 mV	No difference between *Baiap2l2*$^{tm1b/tm1b}$ and control.	*Baiap2l2*$^{tm1b/+}$	0.394	0.119	11	0.0680		*Baiap2l2*$^{tm1b/tm1b}$ vs. *Baiap2l2*$^{tm1b/+}$	t-test	P8-9	8J	
		Baiap2l2$^{tm1b/tm1b}$	0.472	0.094	16							
		Baiap2l2$^{tm1b/+}$	0.360	0.063	5	0.4225		*Baiap2l2*$^{tm1b/tm1b}$ vs. *Baiap2l2*$^{tm1b/+}$		P11		
		Baiap2l2$^{tm1b/tm1b}$	0.398	0.071	4							
14. Uptake of FM1-43 by OHCs	FM1-43 uptake is highly reduced in OHCs from *Baiap2l2*$^{\Delta16/+}$ mice	No BAPTA *Baiap2l2*$^{\Delta16/+}$	100.0	11.9	24	0.0001	%	*Baiap2l2*$^{tm1b/tm1b}$ vs. *Baiap2l2*$^{tm1b/+}$	t-test		8M	
		No BAPTA *Baiap2l2*$^{\Delta16/\Delta16}$	66.4	15.8	36							
		5 mM BAPTA *Baiap2l2*$^{\Delta16/+}$	45.6	11.5	24	0.0001						
		5 mM BAPTA *Baiap2l2*$^{\Delta16/\Delta16}$	37.4	6.1	24							
15. Size and Po of the MET current in adult IHCs	No difference between *Baiap2l2*$^{tm1b/tm1b}$ and control.	*Baiap2l2*$^{tm1b/+}$	-358	96	16	0.286	pA	*Baiap2l2*$^{tm1b/tm1b}$ vs. *Baiap2l2*$^{tm1b/+}$	t-test	P19-P28	8O left panel	
		Baiap2l2$^{tm1b/tm1b}$	-317	84	12							
		Baiap2l2$^{tm1b/+}$	0.023	0.008	16	0.3229					8O right panel	
		Baiap2l2$^{tm1b/tm1b}$	0.026	0.006	12							
16. IHC I_K at 0 mV, $I_{K,f}$ at -25 mV and $I_{K,n}$ at -124 mV	No difference between *Baiap2l2*^{tm1}	*Baiap2l2*$^{tm1b/+}$ I_K	10.06	3.10	7	0.3001	nA	*Baiap2l2*$^{tm1b/tm1b}$ vs. *Baiap2l2*$^{tm1b/+}$	t-test	P28-38	9E, left panel	
		Baiap2l2$^{tm1b/tm1b}$ I_K	11.49	1.99	8							
		Baiap2l2$^{tm1b/+}$ $I_{K,f}$	1.29	0.21	7	0.1312	nA	*Baiap2l2*$^{tm1b/tm1b}$ vs. *Baiap2l2*$^{tm1b/+}$	t-test	P28-38	9E, right panel	
		Baiap2l2$^{tm1b/tm1b}$ $I_{K,f}$	1.53	0.35	8							
		Baiap2l2$^{tm1b/+}$ $I_{K,n}$	0.245	0.052	6	0.8122	nA		t-test	P28-38	9G	

	$^{b/tm1b}$ and control.	*Baiap2l2*$^{tm1b/tm1b}$ $I_{K,n}$	0.235	0.097	7			*Baiap2l2*$^{tm1b/tm1b}$ vs. *Baiap2l2*$^{tm1b/+}$				
		Baiap2l2$^{tm1b/tm1b}$ *Cm*	12.2	2.9	8							
17. OHC $I_{K,n}$ at -124 mV	No difference between *Baiap2l2*$^{tm1b/tm1b}$ and control.	*Baiap2l2*$^{tm1b/+}$ $I_{K,n}$	0.440	0.051	3	0.4592	nA	*Baiap2l2*$^{tm1b/tm1b}$ vs. *Baiap2l2*$^{tm1b/+}$	t-test	P12	10C	
		Baiap2l2$^{tm1b/tm1b}$ $I_{K,n}$	0.410	0.037	3							
		Baiap2l2$^{tm1b/+}$ $I_{K,n}$	0.857	0.220	8	0.1022	nA	*Baiap2l2*$^{tm1b/tm1b}$ vs. *Baiap2l2*$^{tm1b/+}$	t-test	P29-40	10F	
		Baiap2l2$^{tm1b/tm1b}$ $I_{K,n}$	0.671	0.150	6							
