# Supplementary material for: Loss of Baiap2l2 destabilizes the transducing stereocilia of cochlear hair cells and leads to deafness
Source: J Physiol. 2020 Nov 26;599(4):1173–98. doi: 10.1113/JP280670 (PMC7898316; doi:10.1113/JP280670)
Supplement: Supplementary file 2 — Data S1 [file TJP-599-1173-s002.pdf]

Loss of *Baiap2l2* destabilizes the transducing stereocilia of cochlear hair cells and leads to deafness

Adam J. Carlton, Julia Halford, Anna Underhill, Jing-Yi Jeng, Matthew R. Avenarius, Merle L. Gilbert, Federico Ceriani, Kimimuepigha Ebisine, Steve D. M. Brown, Michael R. Bowl, Peter G. Barr-Gillespie, Walter Marcotti

Data points for Figures 2*B,C,F,G*; Figure 8*C,F*; Figure 9C; Figure 10*B,E*.

Figure 2

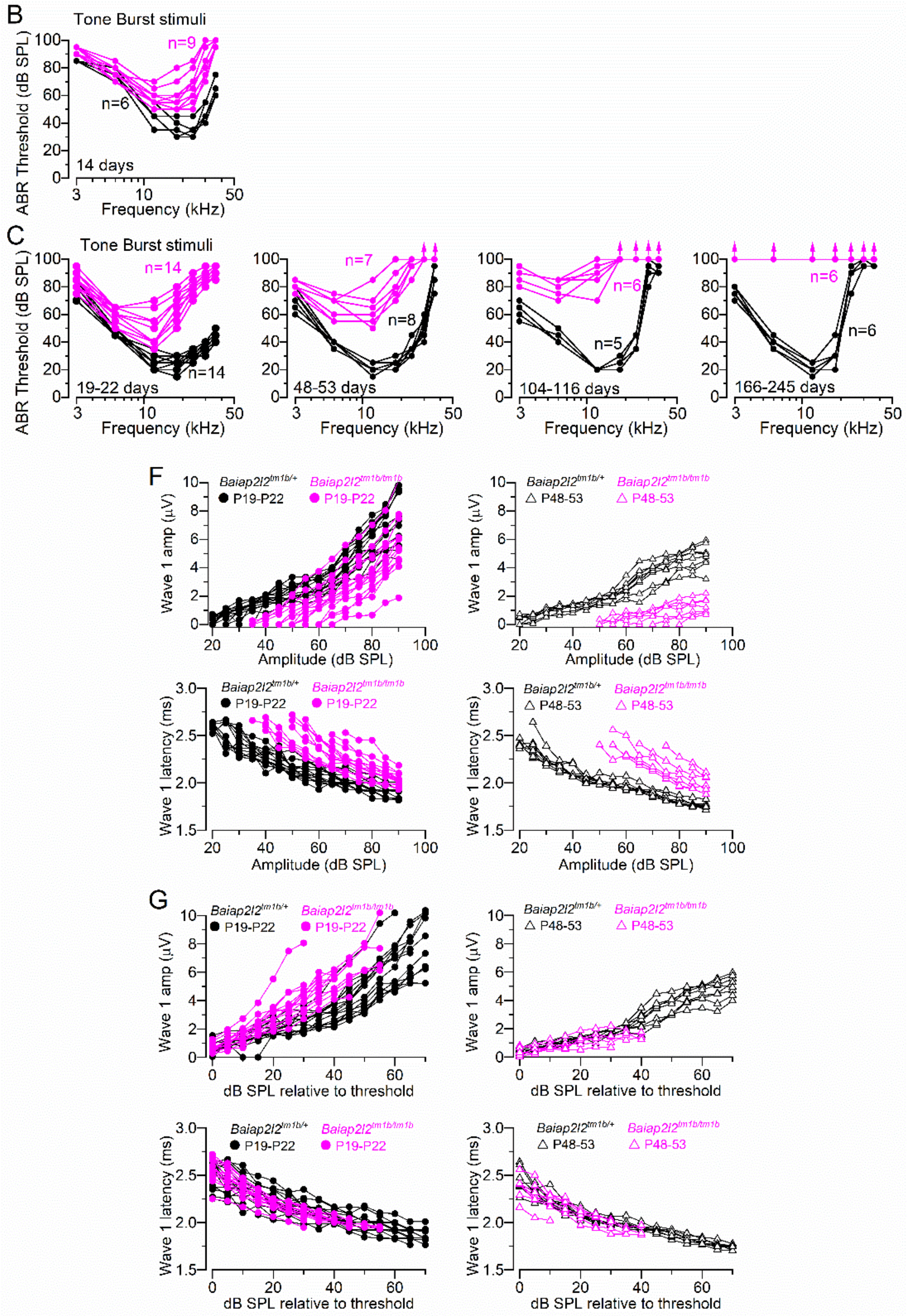

Figure 2: ABR thresholds evoked in *Baiap2l2* mice

Single recordings used to calculate the average values shown in panel ***B***, ***C***, ***F*** and ***G*** of Figure 2 in the original manuscript. Note that due to the large number of recordings for each conditions, data from control (*Baiap2l2*$^{tm1b/+}$, left columns) and knockout (*Baiap2l2*$^{tm1b/tm1b}$, right columns) mice in panel ***F*** and ***G*** have been plotted separately.

Figure 8

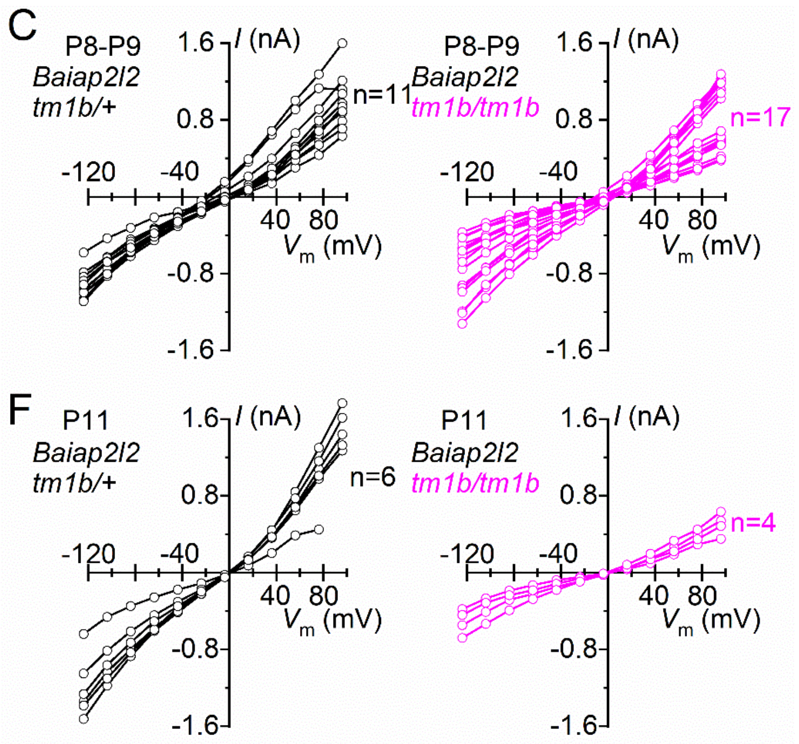

Figure 8. Mechanoelectrical transduction in reduced in *Baiap2l2*^{tmtb} mice

Single recordings used to calculate the average values shown in panel ***C*** and ***F*** of Figure 8 in the original manuscript. Note that due to the large number of recordings for each conditions, data from control (*Baiap2l2*$^{tm1b/+}$, left columns) and knockout (*Baiap2l2*$^{tm1b/tm1b}$, right columns) mice have been plotted separately.

Figure 9

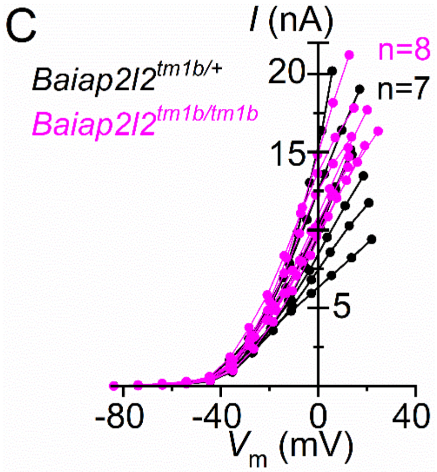

Figure 9. The basolateral membrane properties of adult IHCs are indistinguishable between control and littermate *Baiap2l2$^{tm1b/tm1b}$* mice

Single recordings used to calculate the average values shown in panel ***C*** of Figure 9 in the original manuscript.

Figure 10

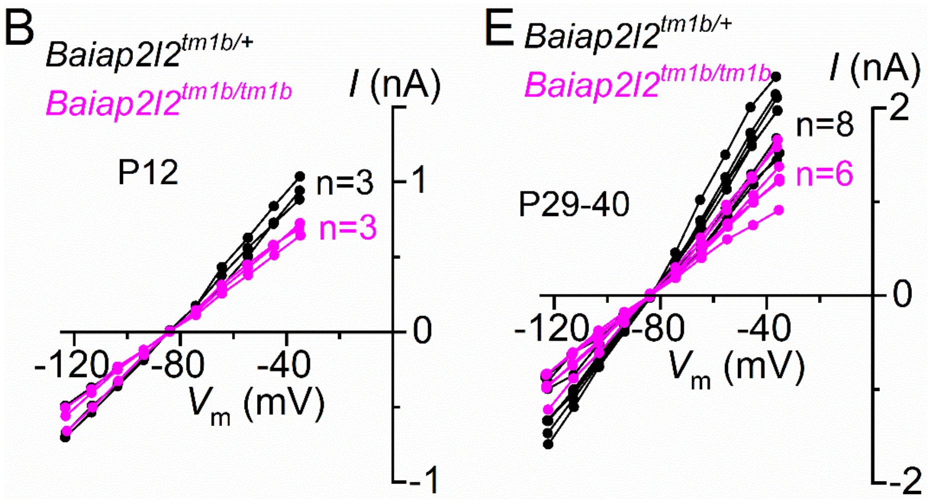

Figure 10. The basolateral membrane properties of adult OHCs are preserved in *Baiap2l2^{tm1b}* mice

Single recordings used to calculate the average values shown in panel ***B*** and ***E*** of Figure 10 in the original manuscript.
